# The impact of COVID-19 on surgical procedures in Japan: analysis of data from the National Clinical Database

**DOI:** 10.1007/s00595-021-02406-2

**Published:** 2021-11-16

**Authors:** Norihiko Ikeda, Hiroyuki Yamamoto, Akinobu Taketomi, Taizo Hibi, Minoru Ono, Naoki Niikura, Iwao Sugitani, Urara Isozumi, Hiroaki Miyata, Hiroaki Nagano, Michiaki Unno, Yuko Kitagawa, Masaki Mori

**Affiliations:** 1 Committee for Novel Coronavirus Disease 2019 Outbreak of Japanese Surgical Society, Tokyo, Japan; 2 Department of Surgery, Tokyo Medical University, 6-7-1 Nishishinjuku, Shinjuku-ku, Tokyo, 160-0023 Japan; 3 Department of Healthcare Quality Assessment, The University of Tokyo, 7-3-1 Hongo, Bunkyo-ku, Tokyo, 113-8655 Japan; 4 Department of Gastroenterological Surgery I, Hokkaido University Graduate School of Medicine, Kita-ku, Kita 15, Nishi 7, Sapporo, Hokkaido 060-8638 Japan; 5 Department of Pediatric Surgery and Transplantation, Kumamoto University Graduate School of Medical Sciences, 1-1-1 Honjo, Chuo-ku, Kumamoto, 860-8556 Japan; 6 Department of Cardiovascular Surgery, Graduate School of Medicine, The University of Tokyo, 7-3-1 Hongo, Bunkyo-ku, Tokyo, 113-8655 Japan; 7 Department of Breast Surgery, Tokai University, School of Medicine, 143, Shimokasuya, Isehara, Kanagawa 259-1193 Japan; 8 Department of Endocrine Surgery, Nippon Medical School Graduate School of Medicine, 1-1-5, Bunkyo-ku, Tokyo, 113-8603 Japan; 9 Department of Gastroenterological, Breast and Endocrine Surgery, Yamaguchi University Graduate School of Medicine, 1-1-1 Minami-Kogushi, Ube, Yamaguchi 755-8505 Japan; 10 Department of Surgery, Tohoku University Graduate School of Medicine, 1-1, Seiryo-machi, Aoba-ku, Sendai, Miyagi 980-8574 Japan; 11 Department of Surgery, Keio University School of Medicine, 35 Shinanomachi, Shinjuku-ku, Tokyo, 160-8582 Japan; 12 School of Medicine, Tokai University, 143, Shimokasuya, Isehara, Kanagawa 259-1193 Japan; 13 President, the Japan Surgical Society, Tokyo, Japan

**Keywords:** COVID-19, Surgical triage, National clinical database

## Abstract

**Background and purpose:**

The spread of COVID-19 has restricted the delivery of standard medical care to surgical patients dramatically. Surgical triage is performed by considering the type of disease, its severity, the urgency for surgery, and the condition of the patient, in addition to the scale of infectious outbreaks in the region. The purpose of this study was to evaluate the impact of the COVID-19 pandemic on the number of surgical procedures performed and whether the effects were more prominent during certain periods of widespread infection and in the affected regions.

**Methods:**

We selected 20 of the most common procedures from each surgical field and compared the weekly numbers of each operation performed in 2020 with the respective numbers in 2018 and 2019, as recorded in the National Clinical Database (NCD). The surgical status during the COVID-19 pandemic as well as the relationship between surgical volume and the degree of regional infection were analyzed extensively.

**Results:**

The rate of decline in surgery was at most 10–15%. Although the numbers of most oncological and cardiovascular procedures decreased in 2020, there was no significant change in the numbers of pancreaticoduodenectomy and aortic replacement procedures performed in the same period.

**Conclusion:**

The numbers of most surgical procedures decreased in 2020 as a result of the COVID-19 pandemic; however, the precise impact of surgical triage on decrease in detection of disease warrants further investigation.

**Supplementary Information:**

The online version contains supplementary material available at 10.1007/s00595-021-02406-2.

## Introduction

The rapid spread of the novel coronavirus disease 2019 (COVID-19), which first appeared in Wuhan, China, evolved into a global pandemic, disrupting all aspects of life across the world, including Japan [1, 2]. The number of infected people has increased dramatically since the first case was reported in Japan on January 15, 2020; however, a comprehensive strategy for the diagnosis and treatment of COVID-19 is yet to be established. Although approximately 80% of patients who acquire COVID-19 recover, there is a high risk of COVID-19 infection resulting in severe disease or death, especially among elderly people with comorbidities such as chronic obstructive pulmonary disease, chronic kidney disease, diabetes, hypertension, cerebrocardiovascular disease, obesity, and people with malignant tumors [3].

Medical resources such as labor, space, and equipment often needed to be reallocated to manage the influx of COVID-19 patients and this restricted the ability to deliver standard medical care to patients with other diseases. However, surgical care should not be interrupted even under such circumstances, as surgeons have multiple responsibilities to continue surgical treatment even in difficult situations such as a pandemic. Nevertheless, surgeons must select which surgical procedures to perform with careful consideration of many factors, ensuring the management of in-hospital surgical systems and preventing nosocomial infections, especially among perioperative patients. Surgical triage should be performed under comprehensive consideration of the type of disease, its severity, the urgency of surgery, and the condition of the patient, as well as the scale of infectious outbreak in the region and the status of medical care provision in the facility [4].

To manage these conditions, the Japan Surgical Society’s “Guidelines for performing surgical triage during the COVID-19 pandemic”, has classified the status of the medical care system into a ‘stable period’ and a ‘restricted period’ [5]. Diseases or indications for surgery have been divided into three levels:

(A) A disease or condition that is nonfatal or does not require urgent medical intervention.

(B) A disease or condition unlikely to be fatal, but is at risk of becoming severe and being potentially fatal.

(C) A disease or condition that may be fatal in a few days or months without any surgical intervention.

If surgical triage is performed with reference to these guidelines, the number of operations should fluctuate and act as an indicator of the impact of COVID-19 infection on surgical treatment. However, given that little is known about the exact number of operations that were cancelled or postponed and which specialties were most affected during this period in Japan, the current survey of major surgical procedures performed in 2020 represents an overview of surgical practices during the COVID-19 pandemic. It will be an important resource for creating a system to allow continuity of surgical treatment in the event of a disaster, such as the outbreak of a new highly transmissible infection.

## Method

The members of the novel coronavirus disease 2019 outbreak committee of the Japan Surgical Society selected 20 of the most common surgical procedures performed in each surgical field.

- Digestive surgery: gastrectomy (including distal gastrectomy, pylorus-preserving gastrectomy and segmental gastrectomy), low anterior resection of the rectum, hepatectomy of one section or more (excluding left lateral section), pancreatoduodenectomy, appendectomy, and cholecystectomy
- Cardiovascular surgery: valve replacement + valve plasty, ascending aorta replacement + aortic arch replacement, coronary artery bypass grafting (CABG), abdominal aorta replacement (below renal artery), ventricular septal defect closure
- General thoracic surgery: lobectomy (+ mediastinal lymph node dissection), resection of a mediastinal tumor
- Breast surgery: total mastectomy, breast-conserving surgery, sentinel node biopsy
- Endocrine surgery: thyroidectomy, parathyroidectomy
- Pediatric surgery (under 16 years of age): inguinal hernia repair, appendectomy

For this study, lobectomy and thoracic aorta replacement refer to pulmonary lobectomy and ascending aorta replacement + aortic arch replacement, respectively.

The primary outcome measure of this study was to identify the impact of the COVID-19 pandemic on surgical care, including any decrease in the number of surgeries. This was analyzed by extracting essential data from the National Clinical Database (NCD). The NCD is a nationwide web-based surgical patient registration system, which enables the collection of data on all surgical procedures performed in Japan, in addition to perioperative factors. More than 14,340,000 procedures, accounting for more than 90% of all surgeries performed in Japan during this period, have been registered by approximately 5,000 hospitals [6, 7]. The NCD constructed software for an Internet-based data collection system and data managers in participating hospitals were responsible for forwarding their data to the NCD office [7]. Using the NCD to investigate all surgeries performed in 2020 is an ideal means to evaluate this extraordinary change in Japanese surgical practices caused by the spread of COVID-19. The total number and change in numbers of each procedure performed in 2020 were analyzed weekly (STATA 17, STATA Corp., TX, USA) and compared with the status in 2018 and 2019.

Some concerns were raised about the possibility of a stronger impact on surgery during certain periods of widespread infection or in areas with high numbers of infected people. The following two settings were used to clarify such speculation.

### Period of COVID-19 pandemic

The first and second waves of the COVID-19 pandemic in Japan were recognized as periods, or phases, when the spread of infection was remarkable. There is no fixed definition for this specific period in the pandemic; therefore, we determined these periods for our study based on changes in the number of infected people nationwide and the objective public view.

The first pandemic wave was stipulated as being from February 26 to May 26, 2020, because the government decided on the basic policy for infection control on February 25 [8] and the state of emergency was lifted nationwide on May 25 [9]. The second wave was from July 1 to September 29, 2020, as the government called for thorough countermeasures to address the increase in the number of infected people on June 30 [10]. The dramatic increase in the number of newly infected people as of September 30 was also the focus of discussion during the Tokyo Metropolitan Coronavirus Infection Monitoring Conference [11].

### Classification of prefectures according to the degree of infection

The cumulative number of infected people per population of prefectures (as of the end of 2020) [12] was used as an index of the degree of infection. Based on this value, the degree of infection in prefectures was classified into three groups: high, medium and low.

- High group: Aichi, Chiba, Fukuoka, Hokkaido, Hyogo, Kanagawa, Kyoto, Nara, Okinawa, Osaka, Saitama, and Tokyo (12 prefectures)
- Medium group: Fukushima, Gifu, Gunma, Hiroshima, Ibaragi, Ishikawa, Kagoshima, Kochi, Kumamoto, Mie, Miyagi, Miyazaki, Nagano, Oita, Okayama, Saga, Shiga, Shizuoka, Tochigi, Toyama, Yamanashi, and Wakayama (22 prefectures)
- Low group: Akita, Aomori, Ehime, Fukui, Iwate, Kagawa, Nagasaki, Niigata, Shimane, Tokushima, Tottori, Yamagata and Yamaguchi (13 prefectures)

We compared the total number of each of the 20 surgical procedures performed in 2019 and 2020 and investigated whether there was a significant decrease in the number of these operations performed in the first and second wave periods in 2020, compared with the same period in 2019.

We also analyzed whether the numbers decreased more significantly in prefectures with higher infection levels throughout the year or during the first and second waves compared with other regions. The two-way repeated-measures analysis of variance (two-way RMANOVA) was used for statistical analysis. The level of statistical significance was set at *p* < 0.05.

## Results

**Fig. 1:**
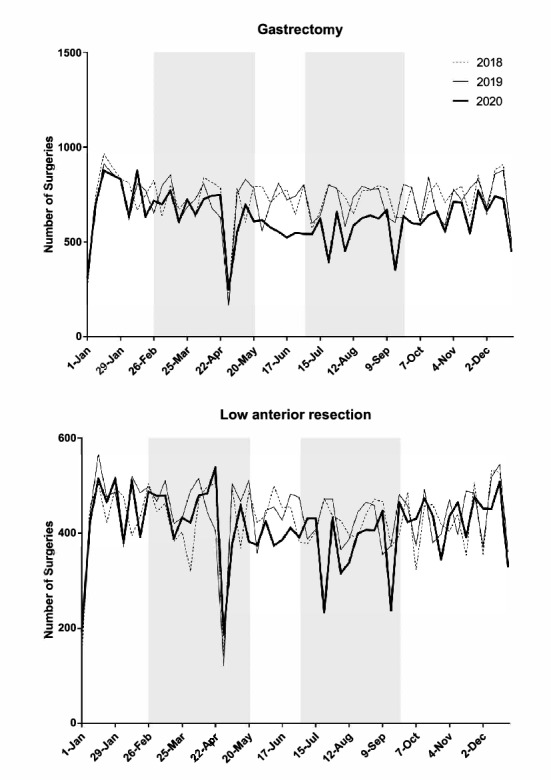

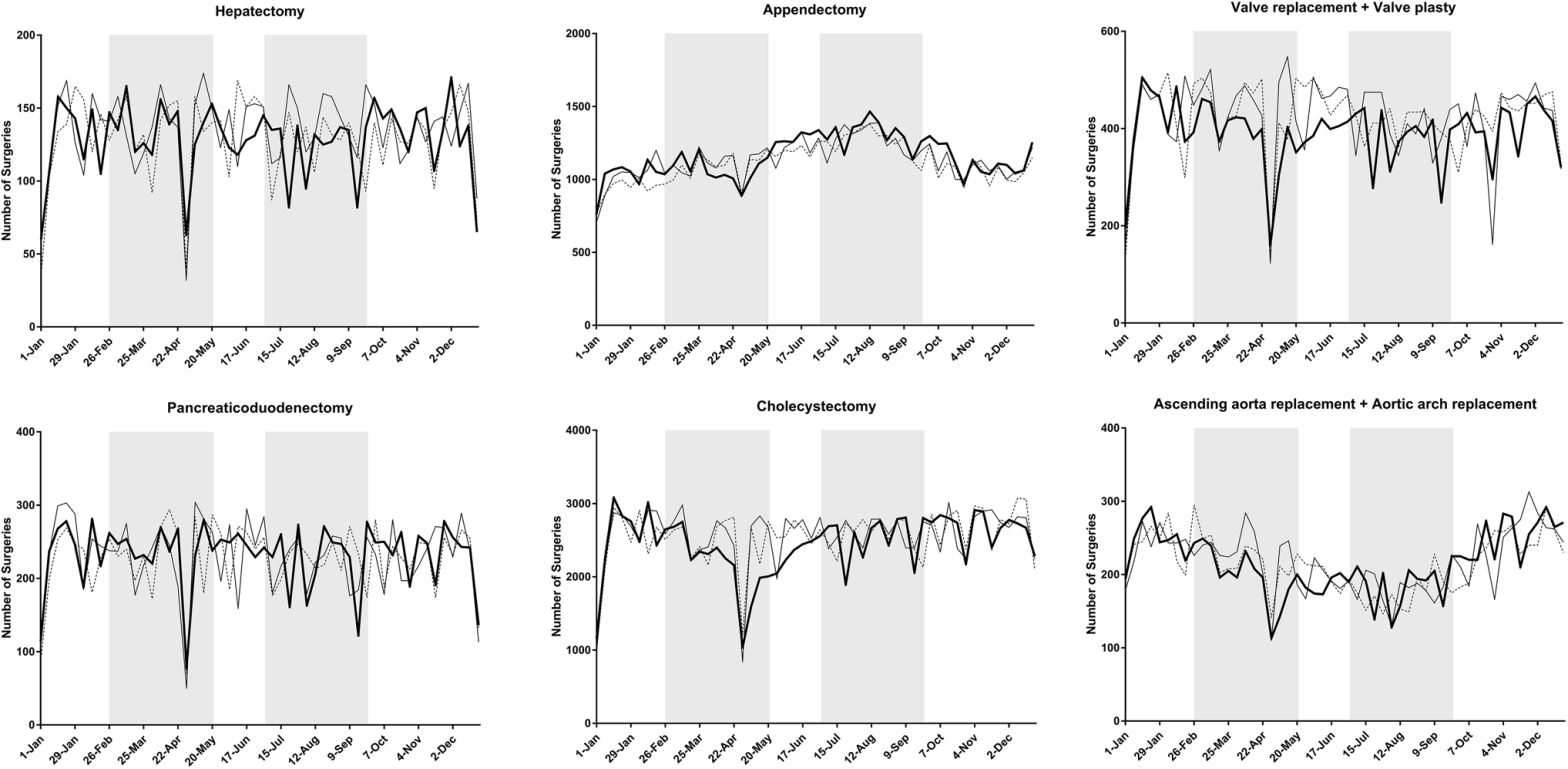

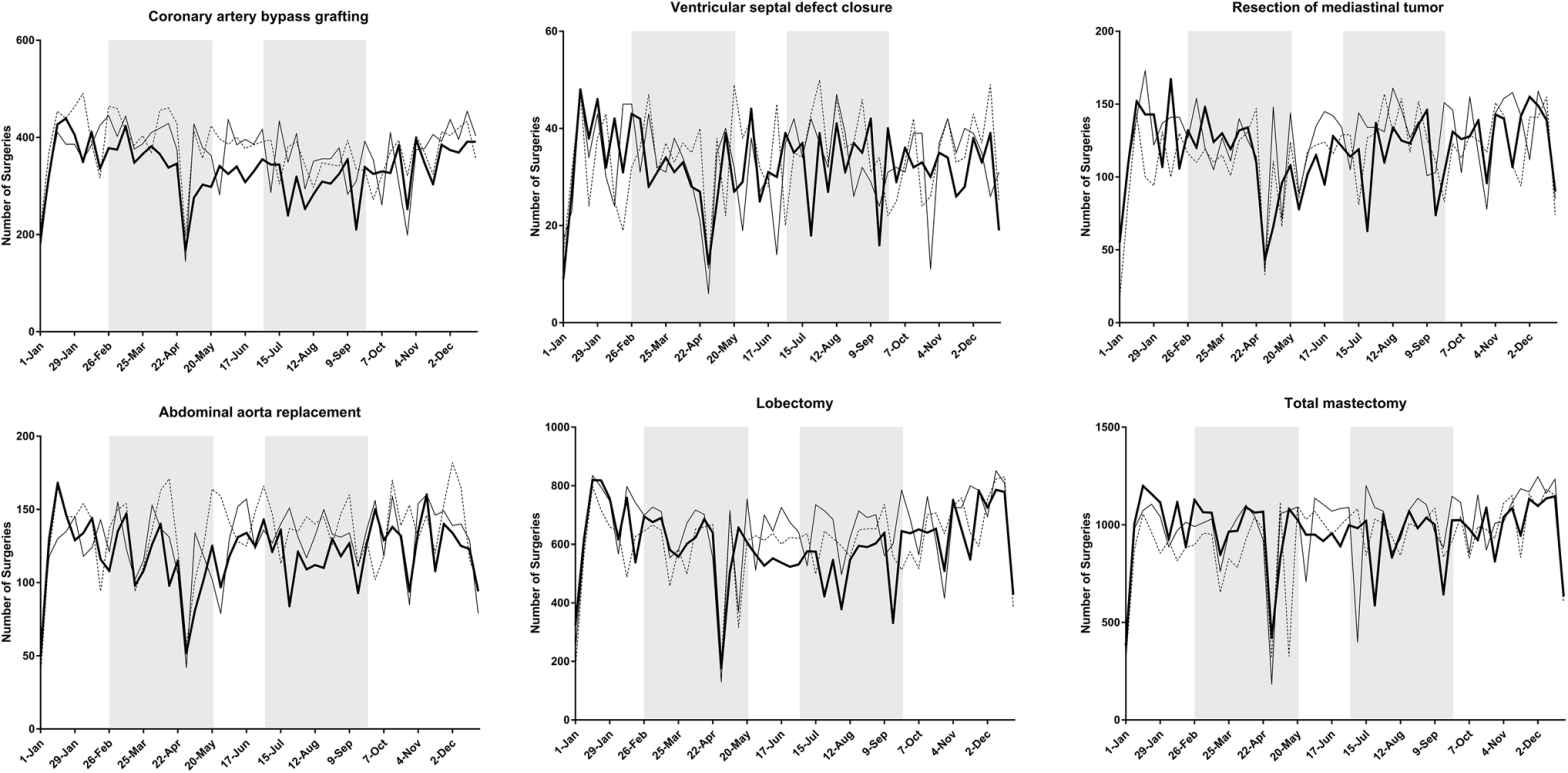

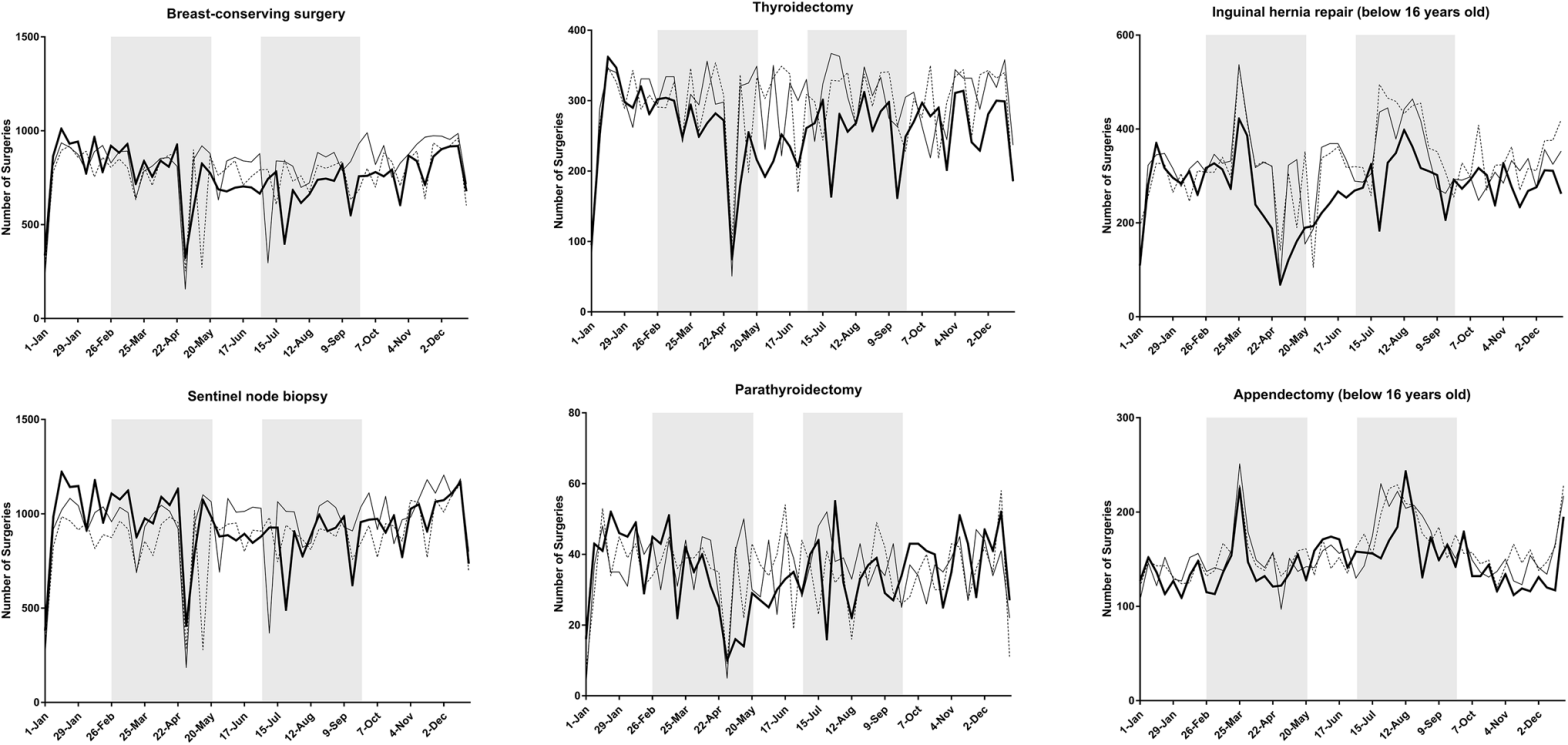
Trends in the weekly volume of each procedure (2018–2020) Shaded areas show the periods of the first and second pandemic waves (February 26-May 26, and July 1-September 29, respectively)

Table 1 summarizes the status of surgery for the 20 procedures**.** A total of 530,701 operations were scheduled between January 1 and December 31, 2020, which corresponded to 95.0% and 97.5% of the total number of surgeries performed in 2018 and 2019, respectively. Cases of unknown age and gender were excluded from the analysis. Figure 1 shows the weekly number of each of the 20 procedures in 2020 as line graphs and the status in 2018 and 2019 for comparison.

**Table 1:** Number of operations performed for each procedure in 2020 vs. 2018 and 2019

Procedure	Number of operations (2018)	Number of operations (2019)	Number of operations (2020)	vs. 2018	vs. 2019
Gastrectomy	37,733	37,173	32,723	86.7%	88.0%
Low anterior resection	22,099	22,763	21,506	97.3%	94.5%
Hepatectomy	6734	7019	6707	99.6%	95.6%
Pancreaticoduodenectomy	11,774	11,963	12,074	102.5%	100.9%
Appendectomy	57,742	59,152	60,094	104.1%	101.6%
Cholecystectomy	132,766	133,441	127,621	96.1%	95.6%
Valve replacement + valve plasty	21,938	21,887	20,355	92.8%	93.0%
Ascending aorta replacement + aortic arch replacement	11,170	11,375	11,186	100.1%	98.3%
Coronary artery bypass grafting	19,704	19,109	17,452	88.6%	91.3%
Abdominal aorta replacement	6985	6624	6249	89.5%	94.3%
Ventricular septal defect closure	1791	1698	1681	93.9%	99.0%
Lobectomy	31,677	33,815	31,174	98.4%	92.2%
Resection of mediastinal tumor	6011	6575	6152	102.3%	93.6%
Total mastectomy	48,276	51,435	50,283	104.2%	97.8%
Breast-conserving surgery	40,003	42,475	39,495	98.7%	93.0%
Sentinel node biopsy	45,501	49,728	48,848	107.4%	98.2%
Thyroidectomy	15,262	15,405	13,449	88.1%	87.3%
Parathyroidectomy	1824	1879	1827	100.2%	97.2%
Inguinal hernia repair (under age 16)	17,171	16,736	14,232	82.9%	85.0%
Appendectomy (under age 16)	8269	8150	7593	91.8%	93.2%
Total	544,430	558,402	530,701	97.5%	95.0%

Table 2 compares the total numbers of each of the 20 surgical procedures during the first and second waves of the COVID-19 pandemic and outlines the differences in surgical situations among the three prefectural groups according to the degree of infection. Detailed results are described below.

**Table 2:** Number of surgeries performed in the pandemic period and regional infection level classifications

Procedure	Period	Infection level	2018	2019	2020	vs 2018	vs 2019	*p* value vs 2019	*p* value high vs medium + low
Gastrectomy	1st pandemic period	High	5176	5166	4676	90.3%	90.5%		
		Medium	2795	2597	2586	92.5%	99.6%		
		Low	1177	1215	1223	103.9%	100.7%		
		Subtotal	9148	8978	8485	92.8%	94.5%	0.084	0.087
	2nd pandemic period	High	5222	5255	4018	76.9%	76.5%		
		Medium	2848	2836	2316	81.3%	81.7%		
		Low	1321	1244	1012	76.6%	81.4%		
		Subtotal	9391	9335	7346	78.2%	78.7%	< 0.001	0.122
	Yearly	Total	37,733	37,173	32,723	86.7%	88.0%	< 0.001	0.046
Low anterior resection	1st pandemic period	High	3193	3404	3255	101.9%	95.6%		
		Medium	1659	1661	1669	100.6%	100.5%		
		Low	630	715	668	106.0%	93.4%		
		Subtotal	5482	5780	5592	102.0%	96.7%	< 0.001	< 0.001
	2nd pandemic period	High	3152	3341	2941	93.3%	88.0%		
		Medium	1617	1558	1428	88.3%	91.7%		
		Low	662	643	567	85.6%	88.2%		
		Subtotal	5431	5542	4936	90.9%	89.1%	0.090	0.003
	Yearly	Total	22,099	22,763	21,506	97.3%	94.5%	< 0.001	< 0.001
Hepatectomy	1st pandemic period	High	999	1017	978	97.9%	96.2%		
		Medium	494	544	544	110.1%	100.0%		
		Low	201	184	213	106.0%	115.8%		
		Subtotal	1694	1745	1735	102.4%	99.4%	0.886	0.333
	2nd pandemic period	High	970	1083	950	97.9%	87.7%		
		Medium	486	568	474	97.5%	83.5%		
		Low	171	169	182	106.4%	107.7%		
		Subtotal	1627	1820	1606	98.7%	88.2%	0.018	0.543
	Yearly	Total	6734	7019	6707	99.6%	95.6%	0.034	0.520
Pancreaticoduodenectomy	1st pandemic period	High	1724	1758	1783	103.4%	101.4%		
		Medium	897	871	919	102.5%	105.5%		
		Low	349	361	342	98.0%	94.7%		
		Subtotal	2970	2990	3044	102.5%	101.8%	0.612	0.970
	2nd pandemic period	High	1685	1791	1748	103.7%	97.6%		
		Medium	892	801	842	94.4%	105.1%		
		Low	341	347	339	99.4%	97.7%		
		Subtotal	2918	2939	2929	100.4%	99.7%	0.935	0.536
	Yearly	Total	11,774	11,963	12,074	102.5%	100.9%	0.627	0.429
Appendectomy	1st pandemic period	High	8609	8753	8268	96.0%	94.5%		
		Medium	3958	4113	4081	103.1%	99.2%		
		Low	1556	1538	1474	94.7%	95.8%		
		Subtotal	14,123	14,404	13,823	97.9%	96.0%	0.038	0.155
	2nd pandemic period	High	9854	9916	10,325	104.8%	104.1%		
		Medium	4704	4874	4920	104.6%	100.9%		
		Low	1828	1694	1801	98.5%	106.3%		
		Subtotal	16,386	16,484	17,046	104.0%	103.4%	0.037	0.326
	Yearly	Total	57,742	59,152	60,094	104.1%	101.6%	0.080	0.808
Cholecystectomy	1st pandemic period	High	17,991	18,290	15,688	87.2%	85.8%		
		Medium	9705	9984	9012	92.9%	90.3%		
		Low	4036	3971	3699	91.7%	93.2%		
		Subtotal	31,732	32,245	28,399	89.5%	88.1%	0.001	0.201
	2nd pandemic period	High	18,806	19,373	18,778	99.9%	96.9%		
		Medium	10,381	10,526	10,276	99.0%	97.6%		
		Low	4223	4029	3975	94.1%	98.7%		
		Subtotal	33,410	33,928	33,029	98.9%	97.4%	0.300	0.734
	Yearly	Total	132,766	133,441	127,621	96.1%	95.6%	0.001	0.337
Valve replacement + valve plasty	1st pandemic period	High	3454	3547	3019	87.4%	85.1%		
		Medium	1553	1557	1418	91.3%	91.1%		
		Low	581	548	501	86.2%	91.4%		
		Subtotal	5588	5652	4938	88.4%	87.4%	0.001	0.066
	2nd pandemic period	High	3353	3370	3085	92.0%	91.5%		
		Medium	1487	1496	1315	88.4%	87.9%		
		Low	525	505	532	101.3%	105.3%		
		Subtotal	5365	5371	4932	91.9%	91.8%	0.068	0.573
	Yearly	Total	21,938	21,887	20,355	92.8%	93.0%	< 0.001	0.047
Ascending aorta replacement + aortic arch replacement	1st pandemic period	High	1859	1833	1633	87.8%	89.1%		
		Medium	747	786	733	98.1%	93.3%		
		Low	283	298	234	82.7%	78.5%		
		Subtotal	2889	2917	2600	90.0%	89.1%	0.008	0.453
	2nd pandemic period	High	1468	1532	1538	104.8%	100.4%		
		Medium	597	616	663	111.1%	107.6%		
		Low	213	218	196	92.0%	89.9%		
		Subtotal	2278	2366	2397	105.2%	101.3%	0.745	0.842
	Yearly	Total	11,170	11,375	11,186	100.1%	98.3%	0.391	0.595
Coronary artery bypass grafting	1st pandemic period	High	3253	3145	2738	84.2%	87.1%		
		Medium	1449	1362	1206	83.2%	88.5%		
		Low	529	495	422	79.8%	85.3%		
		Subtotal	5231	5002	4366	83.5%	87.3%	< 0.001	0.178
	2nd pandemic period	High	2924	2919	2524	86.3%	86.5%		
		Medium	1252	1262	1043	83.3%	82.6%		
		Low	420	435	415	98.8%	95.4%		
		Subtotal	4596	4616	3982	86.6%	86.3%	< 0.001	0.311
	Yearly	Total	19,704	19,109	17,452	88.6%	91.3%	< 0.001	0.196
Abdominal aorta replacement	1st pandemic period	High	943	847	759	80.5%	89.6%		
		Medium	577	539	535	92.7%	99.3%		
		Low	166	153	141	84.9%	92.2%		
		Subtotal	1686	1539	1435	85.1%	93.2%	0.133	0.293
	2nd pandemic period	High	1022	979	868	84.9%	88.7%		
		Medium	601	594	482	80.2%	81.1%		
		Low	178	156	180	101.1%	115.4%		
		Subtotal	1801	1729	1530	85.0%	88.5%	0.006	0.730
	Yearly	Total	6985	6624	6249	89.5%	94.3%	0.003	0.892
Ventricular septal defect closure	1st pandemic period	High	282	253	252	89.4%	99.6%		
		Medium	121	123	116	95.9%	94.3%		
		Low	45	41	32	71.1%	78.0%		
		Subtotal	448	417	400	89.3%	95.9%	0.504	0.555
	2nd pandemic period	High	303	306	271	89.4%	88.6%		
		Medium	118	119	132	111.9%	110.9%		
		Low	46	27	34	73.9%	125.9%		
		Subtotal	467	452	437	93.6%	96.7%	0.673	0.130
	yearly	Total	1791	1698	1681	93.9%	99.0%	0.774	0.353
Lobectomy	1st pandemic period	High	4390	4697	4503	102.6%	95.9%		
		Medium	2049	2235	2214	108.1%	99.1%		
		Low	867	955	978	112.8%	102.4%		
		Subtotal	7306	7887	7695	105.3%	97.6%	< 0.001	< 0.001
	2nd pandemic period	High	4600	4998	4173	90.7%	83.5%		
		Medium	2363	2476	1914	81.0%	77.3%		
		Low	973	1003	895	92.0%	89.2%		
		Subtotal	7936	8477	6982	88.0%	82.4%	< 0.001	< 0.001
	yearly	Total	31,677	33,815	31,174	98.4%	92.2%	< 0.001	< 0.001
Resection of mediastinal tumor	1st pandemic period	High	839	956	872	103.9%	91.2%		
		Medium	412	419	443	107.5%	105.7%		
		Low	157	147	147	93.6%	100.0%		
		Subtotal	1408	1522	1462	103.8%	96.1%	0.528	0.260
	2nd pandemic period	High	984	1055	919	93.4%	87.1%		
		Medium	482	471	430	89.2%	91.3%		
		Low	160	183	156	97.5%	85.2%		
		Subtotal	1626	1709	1505	92.6%	88.1%	0.014	0.386
	Yearly	Total	6011	6575	6152	102.3%	93.6%	0.007	0.109
Total mastectomy	1st pandemic period	High	6679	7415	7609	113.9%	102.6%		
		Medium	3061	3555	3582	117.0%	100.8%		
		Low	1123	1288	1420	126.4%	110.2%		
		Subtotal	10,863	12,258	12,611	116.1%	102.9%	0.270	0.912
	2nd pandemic period	High	7581	7899	7252	95.7%	91.8%		
		Medium	3688	3648	3570	96.8%	97.9%		
		Low	1375	1389	1363	99.1%	98.1%		
		Subtotal	12,644	12,936	12,185	96.4%	94.2%	0.245	0.398
	Yearly	Total	48,276	51,435	50,283	104.2%	97.8%	0.195	0.325
Breast-conserving surgery	1st pandemic period	High	5766	6233	6191	107.4%	99.3%		
		Medium	2708	2874	2863	105.7%	99.6%		
		Low	1024	1064	1078	105.3%	101.3%		
		Subtotal	9498	10,171	10,132	106.7%	99.6%	0.903	0.888
	2nd pandemic period	High	5940	6313	5428	91.4%	86.0%		
		Medium	2797	2916	2492	89.1%	85.5%		
		Low	1053	1041	982	93.3%	94.3%		
		Subtotal	9790	10,270	8902	90.9%	86.7%	0.017	0.457
	Yearly	Total	40,003	42,475	39,495	98.7%	93.0%	< 0.001	0.259
Sentinel node biopsy	1st pandemic period	High	6675	7481	7922	118.7%	105.9%		
		Medium	2905	3325	3478	119.7%	104.6%		
		Low	930	1091	1234	132.7%	113.1%		
		Subtotal	10,510	11,897	12,634	120.2%	106.2%	0.034	0.663
	2nd pandemic period	High	7270	7821	7050	97.0%	90.1%		
		Medium	3142	3290	3034	96.6%	92.2%		
		Low	1034	1058	1102	106.6%	104.2%		
		Subtotal	11,446	12,169	11,186	97.7%	91.9%	0.142	0.396
	Yearly	Total	45,501	49,728	48,848	107.4%	98.2%	0.339	0.336
Thyroidectomy	1st pandemic period	High	2267	2311	1887	83.2%	81.7%		
		Medium	1084	1192	1087	100.3%	91.2%		
		Low	343	299	271	79.0%	90.6%		
		Subtotal	3694	3802	3245	87.8%	85.3%	< 0.001	0.010
	2nd pandemic period	High	2353	2481	1911	81.2%	77.0%		
		Medium	1218	1228	1170	96.1%	95.3%		
		Low	347	318	281	81.0%	88.4%		
		Subtotal	3918	4027	3362	85.8%	83.5%	< 0.001	< 0.001
	Yearly	Total	15,262	15,405	13,449	88.1%	87.3%	< 0.001	< 0.001
Parathyroidectomy	1st pandemic period	High	270	273	222	82.2%	81.3%		
		Medium	144	154	143	99.3%	92.9%		
		Low	46	39	38	82.6%	97.4%		
		Subtotal	460	466	403	87.6%	86.5%	0.095	0.292
	2nd pandemic period	High	272	301	262	96.3%	87.0%		
		Medium	125	158	145	116.0%	91.8%		
		Low	45	29	30	66.7%	103.4%		
		Subtotal	442	488	437	98.9%	89.5%	0.204	0.496
	Yearly	Total	1824	1879	1827	100.2%	97.2%	0.477	0.241
Inguinal hernia repair (under age 16)	1st pandemic period	High	2426	2409	1801	74.2%	74.8%		
		Medium	1307	1269	1059	81.0%	83.5%		
		Low	430	448	361	84.0%	80.6%		
		Subtotal	4163	4126	3221	77.4%	78.1%	< 0.001	0.145
	2nd pandemic period	High	2818	2718	2252	79.9%	82.9%		
		Medium	1494	1405	1225	82.0%	87.2%		
		Low	616	512	441	71.6%	86.1%		
		Subtotal	4928	4635	3918	79.5%	84.5%	0.002	0.301
	Yearly	Total	17,171	16,736	14,232	82.9%	85.0%	< 0.001	0.045
Appendectomy (under age 16)	1st pandemic period	High	1192	1155	1066	89.4%	92.3%		
		Medium	602	582	557	92.5%	95.7%		
		Low	223	243	187	83.9%	77.0%		
		Subtotal	2017	1980	1810	89.7%	91.4%	0.009	0.895
	2nd pandemic period	High	1391	1379	1291	92.8%	93.6%		
		Medium	732	719	614	83.9%	85.4%		
		Low	295	261	270	91.5%	103.4%		0.936
		Subtotal	2418	2359	2175	90.0%	92.2%	0.076	0.936
	Yearly	Total	8269	8150	7593	91.8%	93.2%	< 0.001	0.679

### Comparison between 2019 and 2020

#### Digestive surgery

We did not identify a significant change in the number of pancreaticoduodenectomies or appendectomies from 2019; however, the numbers of gastrectomy, low anterior resection of the rectum, hepatectomy, and cholecystectomy, decreased significantly. Among these, the rate of decline in gastrectomies and low anterior resections of the rectum was more prominent in prefectures with high infection levels than in those with moderate or low infection levels. (*p* < 0.001).

#### Cardiovascular surgery

The numbers of thoracic aorta replacement and ventricular septal defect closure procedures did not change significantly from 2019, but the numbers of other procedures, such as valve replacement + valve plasty, CABG and abdominal aorta replacement, decreased significantly (*p* < 0.001, *p* < 0.001, *p* = 0.003, respectively). The rate of decline of CABG and abdominal aorta replacements did not differ by region.

#### General thoracic surgery

The numbers of lobectomy and resection of mediastinal tumors in 2020 decreased from the previous year (*p* < 0.001, both) and the rate of decrease in lobectomies was more significant in prefectures with high infection levels (*p* < 0.001).

#### Breast surgery and endocrine surgery

The operative status for total mastectomy and parathyroidectomy was not significantly different from that in 2019. The number of breast-conserving surgeries and thyroidectomies was significantly lower in 2020 (*p* < 0.001, both) and the rate of decrease for the latter was more prominent in prefectures with high infection levels.

#### Pediatric surgery

The numbers of inguinal hernia repairs and appendectomies were significantly lower in 2020. The rate of decrease in inguinal hernia repairs performed was more pronounced in prefectures with high infection levels, but the rate of decrease in the numbers of appendectomies performed did not differ by region.

### Period of COVID-19 pandemic

**Fig. 2:**
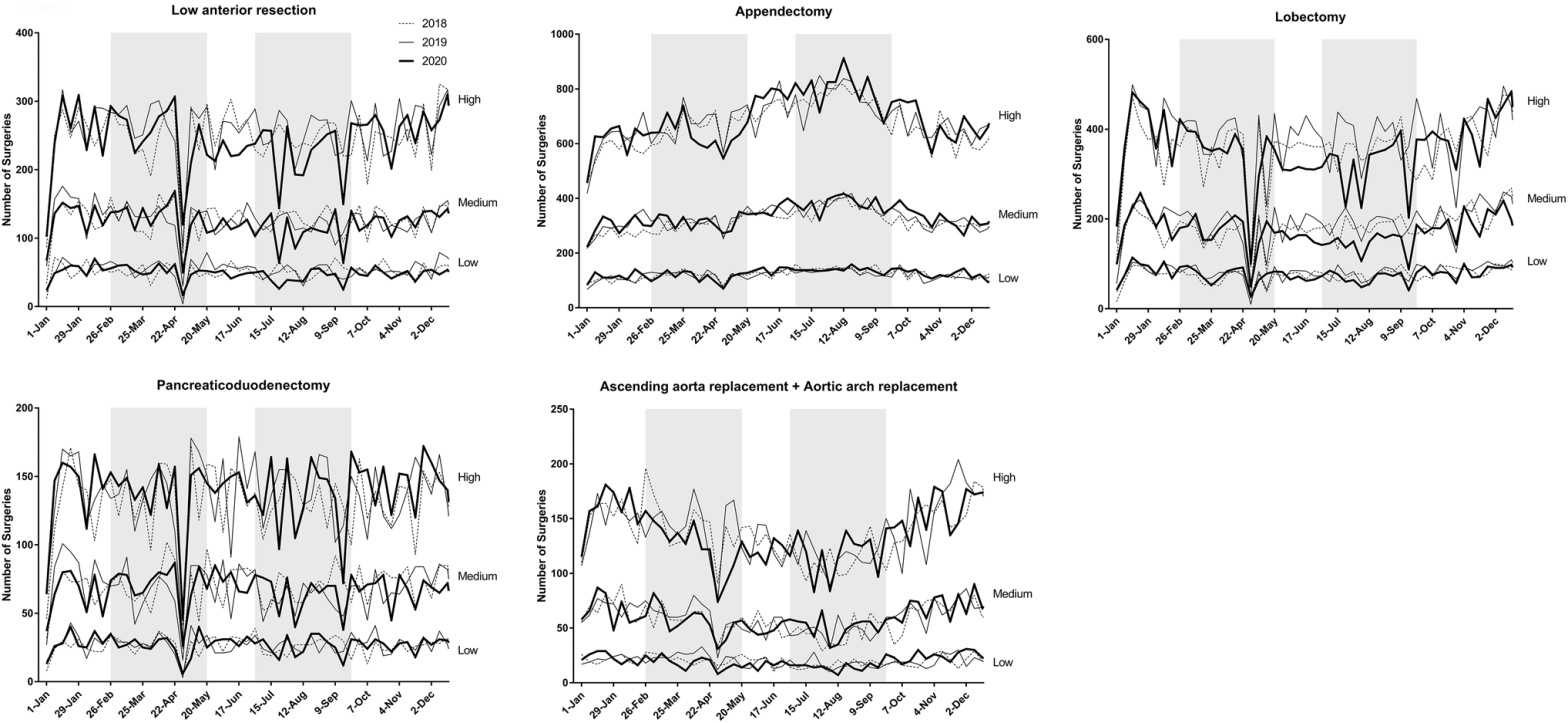
Weekly volume of five procedures according to the three groups (high, medium, low) of regional infection level. Shaded areas show the periods of the first and second pandemic waves (February 26-May 26, and July 1-September 29, respectively)

There was a marked decrease in the numbers of low anterior resection of the rectum, lobectomy of the lung **(**Fig. 2**)** and thyroidectomy in prefectures with high rates of infection compared with the numbers in other areas during the first and second waves of the pandemic in 2020. On the other hand, no such regional differences were evident for pancreaticoduodenectomy, appendectomy, thoracic aorta replacement **(**Fig. 2**)**, VSD closure, total mastectomy, or parathyroidectomy. (Supplemental Fig. 1 shows representative graphs for the other 15 procedures).

## Discussion

We conducted this study to establish the impact of the COVID-19 pandemic on the number of surgical procedures performed in Japan during this period by comparing the total number of operations for 20 representative surgical procedures in 2020 with that of those in the 2 pre-pandemic years. We also evaluated whether the effects of COVID-19 were more serious during certain periods and in regions where the infection was more widespread.

Although the numbers of most operative procedures decreased in 2020, we were able to identify differences in the rate of decline in the numbers for each procedure and evaluate the impact of the scale of infection on surgical treatment. Since the purpose of this study was to provide an overview of surgical procedures in 2020, we did not investigate details such as preoperative disease status, stage of malignancy, or postoperative course. In addition to surgical triage, the decrease in the numbers of surgeries performed may be attributable to multiple factors, such as fewer new patients and postponement of examinations, which will also be the subject of our next study.

Ultimately, surgery should be performed as usual and without delay for symptomatic advanced cancer, although non-aggressive cancers differentiated by improved diagnostic methods may be postponed until the pandemic subsides [4, 13]. Moreover, for high-risk patients with multiple comorbidities, postponing surgery may be necessary to avoid the risk of postoperative infection.

According to a large-scale surgical triage survey of 359 hospitals in 71 countries, including Japan, 73% (approximately 1.4 million) of operations, including upper and lower gastrointestinal, hepatobiliary, urological, head and neck, gynecological, plastic, orthopedic, and obstetric operations, which were scheduled to take place over a 12-week period, from late March 2020, were cancelled or postponed. Among these, approximately 98,000 were procedures for cancer (30%) and 1,253,000 were procedures for benign diseases (84%) [14]. An international, prospective, cohort study including 20,006 patients from 466 hospitals in 61 countries was conducted to reveal the effect of COVID-19 pandemic lockdowns on elective cancer surgery. Of eligible patients awaiting surgery, 0.6% had their surgery postponed during light restrictions, 5.5% in moderate lockdowns, and 15.0% in full lockdowns [15]. We want to entrust further analysis of clinical factors, disease severity, stage, and histological type of malignant tumors and postoperative course to the research in each specialized surgical field in the near future.

Although our study was limited to typical surgical procedures, the results showed that the rate of decline for surgery was no more than 10–15%. It is possible that since Japan had far fewer patients with COVID-19 infection than most Western countries, surgeons could schedule high-priority operations even during the pandemic when the number of COVID-19 cases was growing. Okuno used the Diagnosis Procedure Combination (DPC) data from the Quality Indicator/Improvement Project (QIP) database to compare surgical volume between the two periods of July 2018–March 2020 and April–June 2020. That analysis revealed that the decline in oncological procedures for gastrointestinal, hepato-pancreato-biliary, lung, breast, and genitourinary cancer was not significant, even though the numbers were lower than for the same period in the previous year [16]. Miyawaki compared the number of surgeries across specialties in weeks 2 to 9 versus weeks 10 to 17 in 2020 using the de-identified hospital administrative database from Japanese acute-care hospitals. The rates of decline in cardiovascular surgery and gastrointestinal or hepato-pancreato-biliary system were 9.9% and 9.5%, respectively; however, the number of breast operations did not decrease significantly [17]. There are variations in the trends of the affected number of surgical procedures based on the region and time period [18]. Our study shows that the numbers of low anterior resections of the rectum, CABG, lung lobectomies, thyroidectomies, and inguinal hernia repairs were significantly lower during the first and second COVID-19 pandemic waves. There are various reasons for each disease, such as surgical triage, fewer new patients, and implementation of alternative treatments.

Patients with potentially curable pancreatic carcinoma, pancreatic cystic lesions with confirmed high-grade dysplasia, duodenal cancer, ampullary cancer, as well as curable hepatocellular carcinoma (HCC), and cholangiocarcinoma should undergo surgical resection even during a pandemic [19]. Our results showed that the number of pancreaticoduodenectomies did not decline in 2020, in accordance with consensus statements supported by seven international pancreatic associations [20]. However, other surgical procedures for gastric, colon, liver, lung, and breast cancers, decreased significantly. Another possible cause, along with the impact of triage, may be the 30% decrease in the number of people being screened for cancer in Japan in 2020 [21]. For hepatic malignancies, such as HCC, treating practitioners may select alternative procedures, including radiofrequency ablation and transarterial chemoembolization as locoregional therapies, and molecular targeting drugs for the advanced disease instead of resection for some patients [22].

During the COVID-19 pandemic era, opportunities for cancer screening by upper gastrointestinal endoscopy (UGI) or colonoscopy may have decreased because it is an aerosol-generating procedure [23]. In fact, it was reported that the total volume of endoscopic procedures decreased by 44% during this time [24]. The decline in the number of gastrectomies or anterior resections of the rectum may be related to the decrease in these endoscopic screening procedures.

However, early cancer that is left unscreened might be detected as advanced cancer in the future. Further detailed studies for each cancer could help to verify whether there is a stage shift in surgical cases in the next few years. The significant decrease in the number of cholecystectomies in the present study is in line with an international survey including 14 countries [25], where the majority (72%) of hepato-pancreato-biliary surgeons reported an “alarming decrease” in the number of cholecystectomies during the pandemic. An increase in non-surgical treatment for acute cholecystitis was also reported by multicenter studies from the U.K. and Spain [26, 27], although a multisocietary position statement concluded that laparoscopic cholecystectomy remains the treatment of choice for acute cholecystitis even during the COVID-19 pandemic [28]. Whether the choice of non-surgical treatment for complicated gallstone disease negatively impacted the outcomes of patients warrants further investigation.

Cardiovascular surgery frequently requires transfer of the patient to an intensive care unit and ventilatory support in the postoperative period. However, if there are many patients with respiratory failure caused by COVID-19 pneumonia in the same region, the resources related to intensive care must be reallocated and there may be situations where surgery is limited to life-threatening emergencies. In the United States, there was a 53% reduction in adult cardiac surgeries nationwide during the early half of 2020 compared with 2019, with a 65% decrease in elective surgical cases and a 40% decrease even in non-elective cases [29]. Our results showed that the number of aortic surgeries was the same as in pre-pandemic years because the urgent intervention was required. A global survey of cardiac surgery centers was conducted among the 61 participating centers of the Randomization of Single vs Multiple Arterial Grafts (ROMA) trial, 60 of which responded: 7 from Asia, 2 from Australia, 31 from Europe, 16 from North America, and 4 from South America. The Survey revealed a greater than 50% reduction in ICU bed availability for cardiac surgery and a median reduction in cardiac surgery case volume of 50% to 75% [30].

The number of CABG procedures decreased significantly in 2020, but this could be due to a decrease in the number of new patients or the possibility that catheter-based treatment was performed instead of surgery. The rate of total mastectomies for breast cancer decreased by only about 6% at the time of the second wave, but there was a 13% reduction in breast-conserving surgery. Possible reasons for this include the avoidance of postoperative radiotherapy during the pandemic, or fewer new patients being referred for surgery due to refrained hospital visits. The number of thyroidectomies also decreased in 2020, especially in prefectures with high infection levels, probably because elective surgeries were reserved for patients with very-low risk differentiated thyroid carcinoma or indeterminate thyroid nodules [31].

Most appendectomies are emergency procedures; hence, the numbers of appendectomies in 2020 and 2019 were similar. The number of appendectomies in children decreased, probably due to triage or selection of conservative treatment. Our speculation is supported by a report from a tertiary hospital in New York State, the epicenter of the pandemic in the U.S., where it expanded inclusion criteria for non-operative management of acute appendicitis to reduce operating room utilization [32]. It is noteworthy that multiple publications have described increased incidences of complicated appendicitis during the outbreak [33, 34]. A recent cross-sectional retrospective study based on the Pediatric Health Information System in the U.S. collected data for all patients diagnosed with appendicitis from 52 children’s hospitals between 2017 and 2020 (*n* = 19,431). That study concluded that the increased proportion of complicated appendicitis presentations by 4.4% (from 46.5% to 50.9%) during the COVID-19 pandemic was driven by a decrease in uncomplicated appendicitis [35]. Whether the difference in presentation and management of pediatric appendicitis has resulted in inferior clinical outcomes is subject to further investigation. It will be necessary to investigate the number of operations postponed or canceled. It is also an important issue to proceed with a fact-finding survey on how many patients were disadvantaged by delayed surgery or by receiving alternative treatment.

It is known that perioperative infection is highly likely to cause severe disease. An analysis of 1128 patients (94 with preoperative infection) who were confirmed to be positive for novel coronavirus in the perioperative period revealed a very high 30-day postoperative mortality rate of 23.8% (268 patients), with 81.7% of these patients dying of pulmonary complications [36]. Moreover, 15 (44%) of the 34 patients with confirmed infection required ICU management, with a postoperative mortality rate of 20.5% [37]. Osorio J, et al. also reported that COVID-19 positive patients who underwent emergency general and gastrointestinal surgery during the pandemic had more complications and a higher likelihood of failure rescue than COVID-19 negative patients [38].

It should be noted that the rate of asymptomatic patients diagnosed as positive for infection by PCR testing was reported to range from 6.3% to 91.7% [39]; therefore, asymptomatic infected patients cannot be screened by examination alone, which may lead to serious postoperative complications and consequent nosocomial infections. It is necessary to verify whether the status of postoperative complications in surgical patients differs from that in previous years and to identify complications strongly related to COVID-19 infection.

Since May 2020, the cost of preoperative PCR-based screening for infection has been covered by insurance [40] and is expected to contribute to the recovery of surgical volumes. According to a questionnaire survey conducted by the Japan Surgical Society, 41.7% of all facilities performed PCR testing on patients scheduled for operations, and the implementation of this increased from 23.8% in April 2020 to 54.4% in December 2020 [41]. In the future, it will be necessary to flexibly update surgical treatment algorithms in view of the generalization of preoperative PCR testing and the increased vaccination status of the general population.

In conclusion, this real-world data analysis of surgeries based on NCD data could provide an objective picture of the status of surgical treatment under COVID-19 infection. Although a decrease in the numbers of each surgical procedure during the COVID-19 pandemic is evident, more detailed studies are needed to demonstrate the difference in management according to the severity of disease and the condition of the patient. There are multiple causes for the decline in the number of surgeries, including triage, fewer new patients, and postponement of examinations. An evaluation of the impact of these factors should be performed as the next step of the analysis. We hope that the findings of our study will contribute to even better infection control, strengthen the intensive care system, and secure medical resources to enable a sustainable medical supply system in the event of a pandemic.

## Supplementary Information

Below is the link to the electronic supplementary material.

Supplementary file1 Weekly volume of 15 procedures according to the three groups (high, medium, low) of regional infection level. Shaded areas show the periods of the first and second pandemic waves (February 26-May 26, and July 1-September 29, respectively) (PDF 7242 KB)
