# Supplementary material for: The impact of COVID-19 on surgical procedures in Japan: analysis of data from the National Clinical Database
Source: Surg Today. 2021 Nov 16;52(1):22–35. doi: 10.1007/s00595-021-02406-2 (PMC8592826; doi:10.1007/s00595-021-02406-2)
Supplement: Supplementary file 1 — Supplementary file1 Weekly volume of 15 procedures according to the three groups (high, medium, low) of regional infection level. Shaded areas show the periods of the first and second pandemic waves (February 26-May 26, and July 1-September 29, respectively) (PDF 7242 KB) [file 595_2021_2406_MOESM1_ESM.pdf]

Supplemental Figure 1

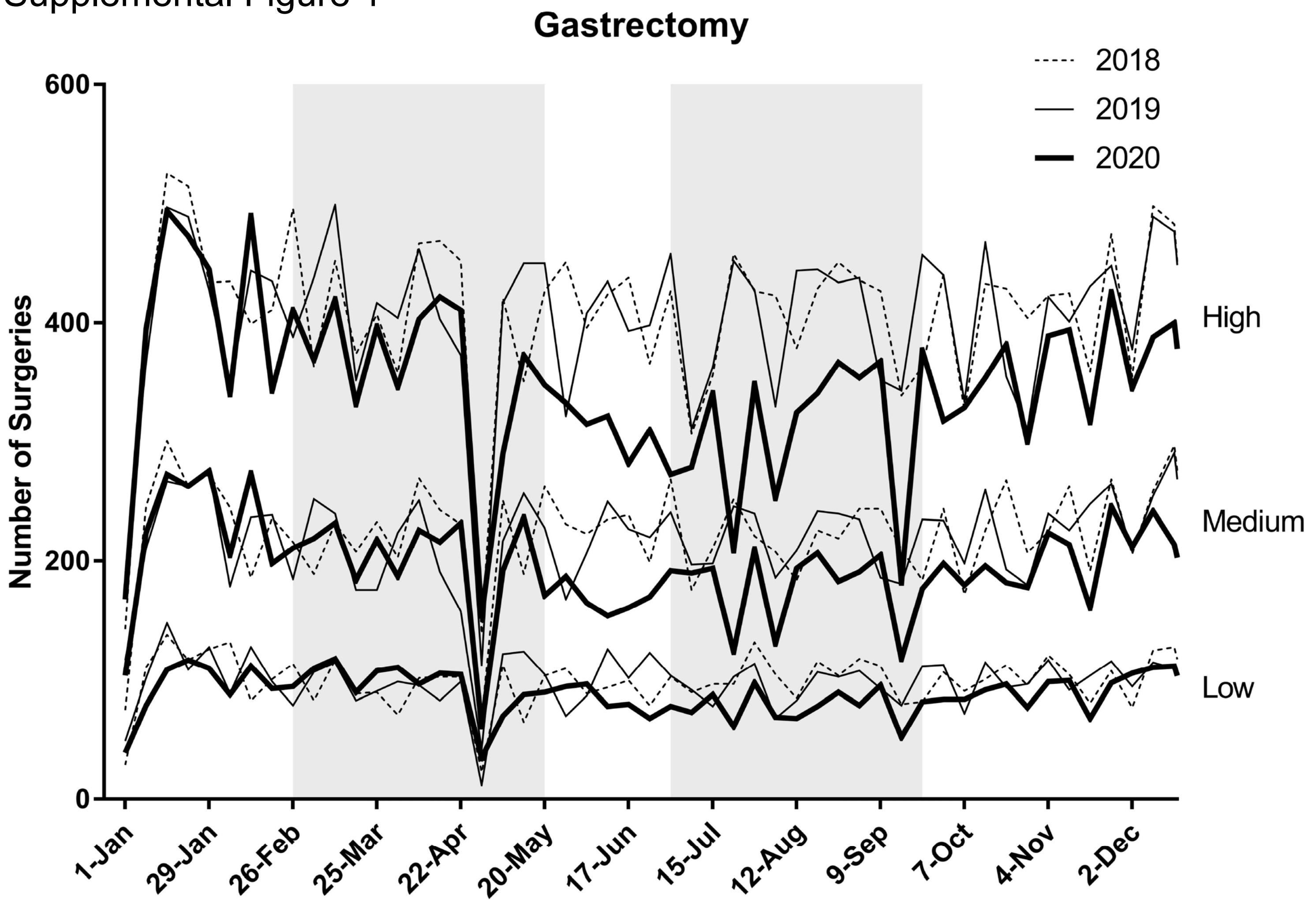


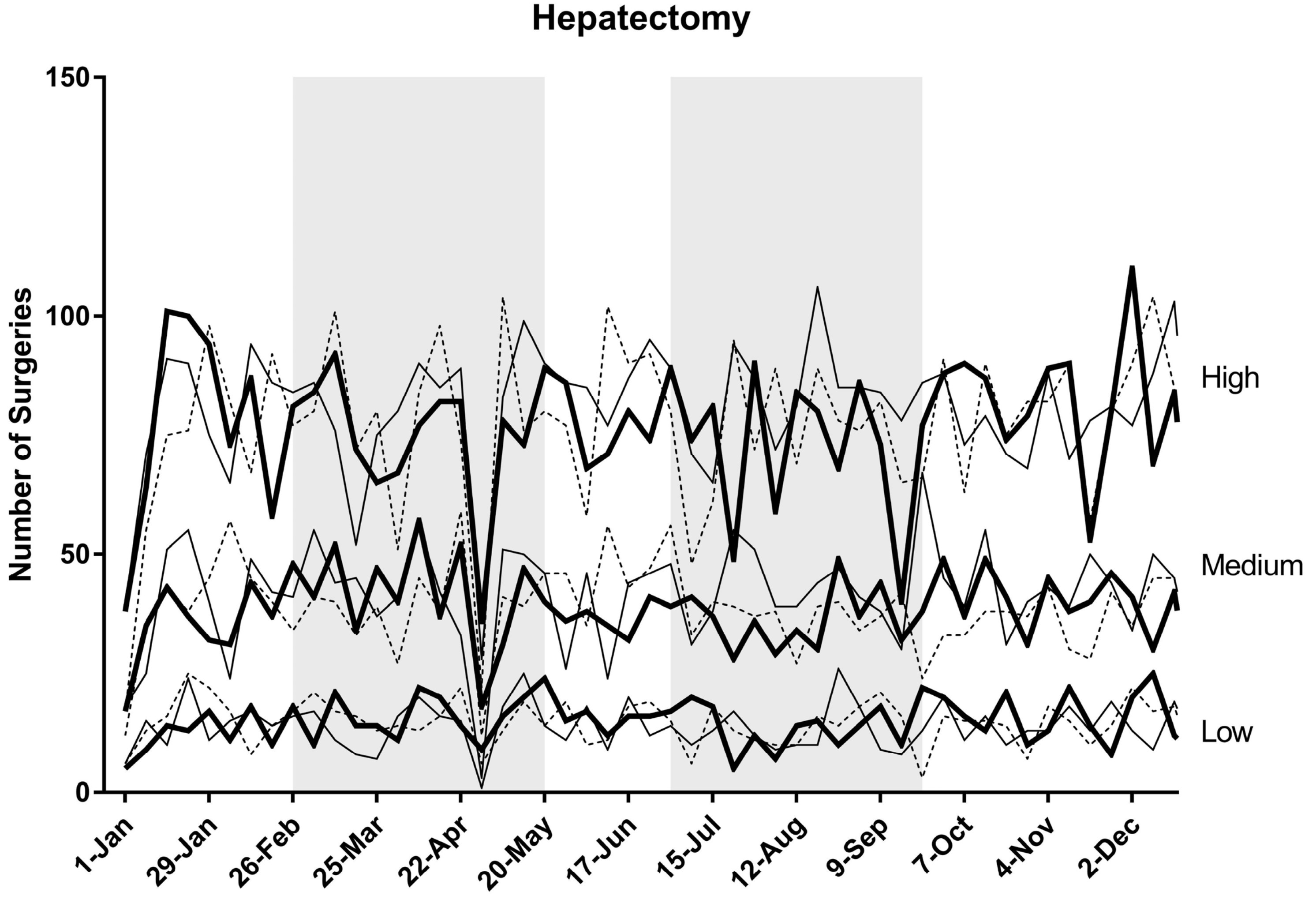

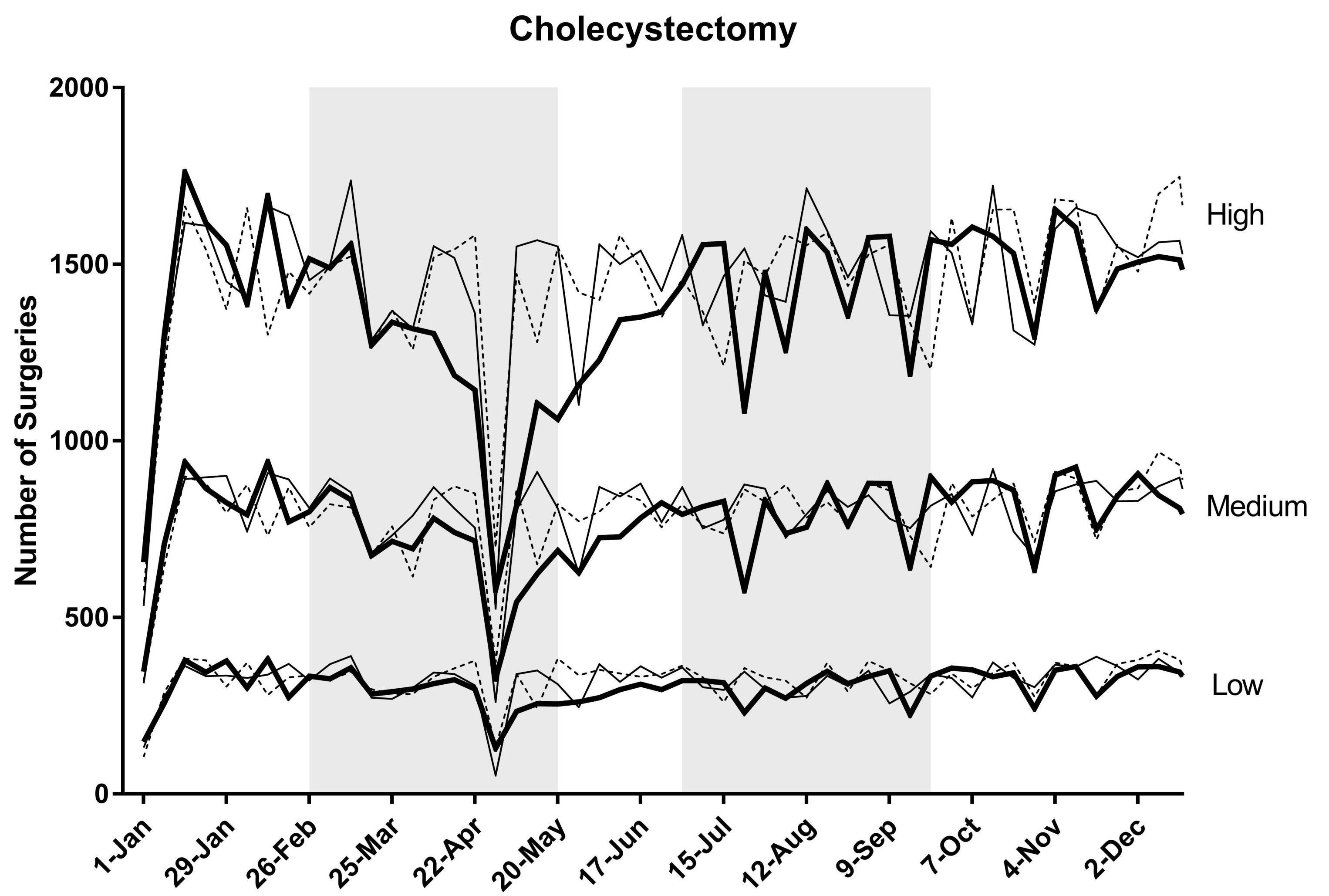

Cholecystectomy
Number of Surgeries
2000
1500
1000
500
0
High
Medium
Low
1-Jan
29-Jan
26-Feb
25-Mar
22-Apr
20-May
17-Jun
15-Jul
12-Aug
9-Sep
7-Oct
4-Nov
2-Dec


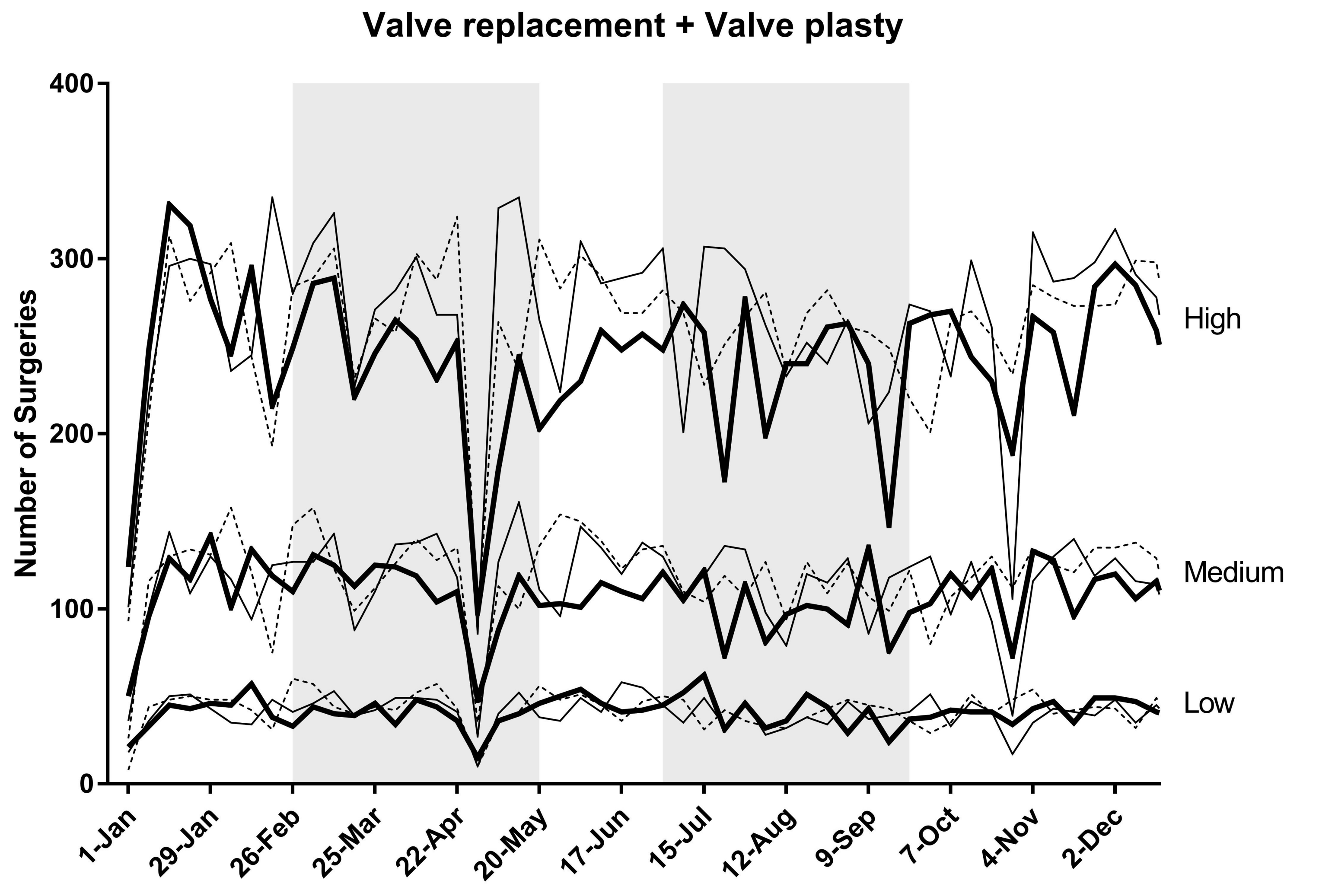

Valve replacement + Valve plasty
Number of Surgeries
400
300
200
100
0
High
Medium
Low
1-Jan
29-Jan
26-Feb
25-Mar
22-Apr
20-May
17-Jun
15-Jul
12-Aug
9-Sep
7-Oct
4-Nov
2-Dec

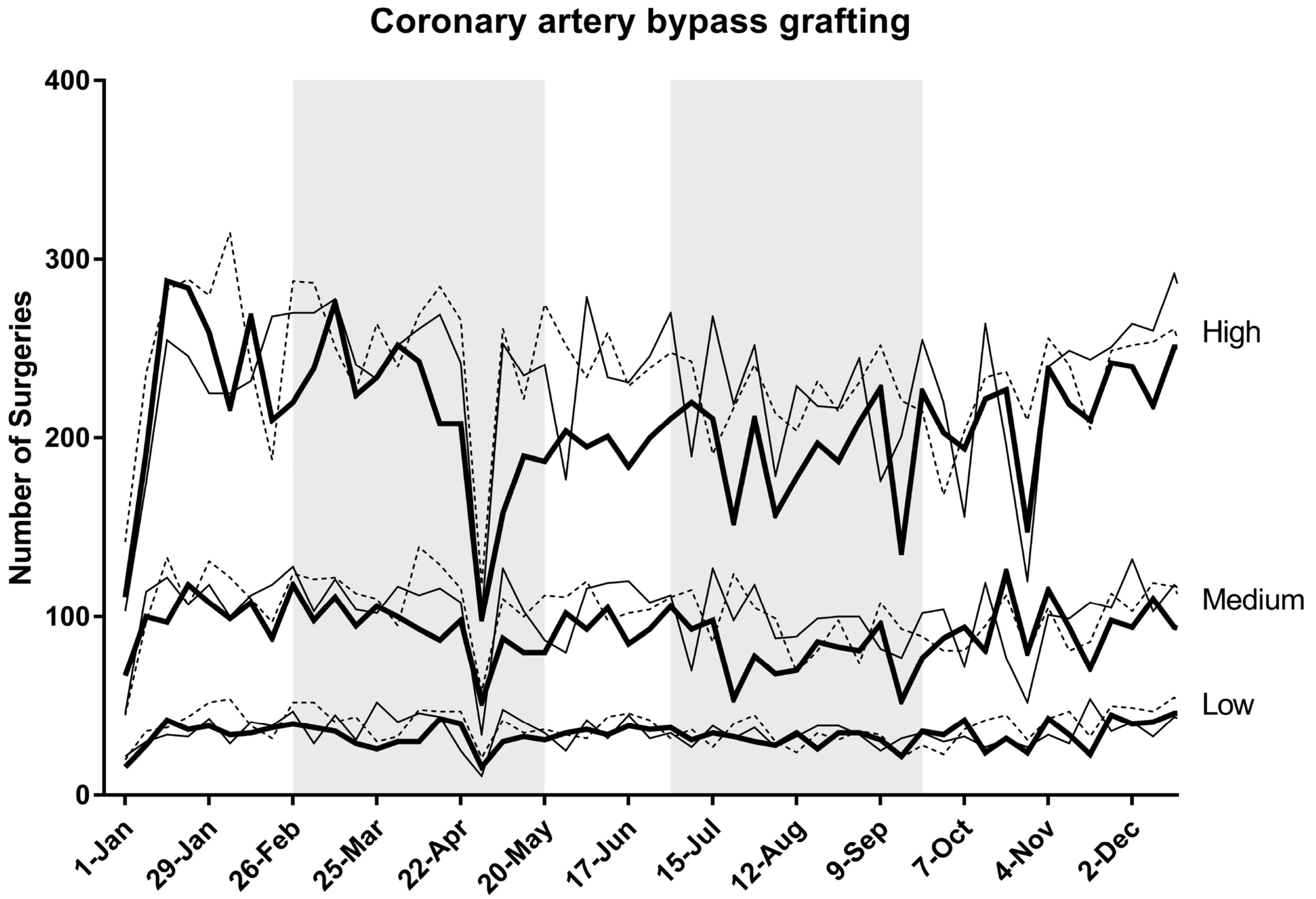
Coronary artery bypass grafting
Number of Surgeries
400
300
200
100
0
High
Medium
Low
1-Jan
29-Jan
26-Feb
25-Mar
22-Apr
20-May
17-Jun
15-Jul
12-Aug
9-Sep
7-Oct
4-Nov
2-Dec

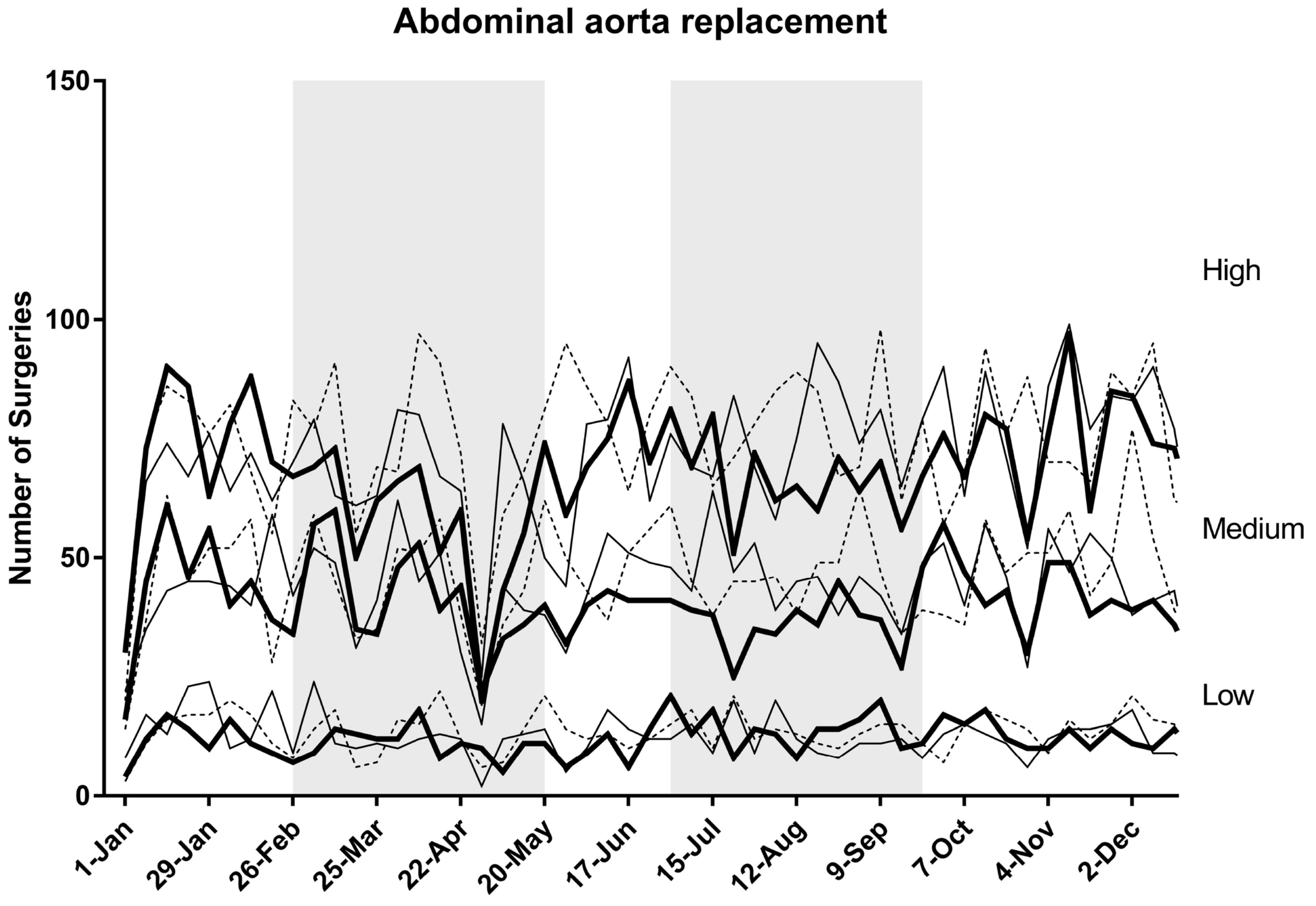
Abdominal aorta replacement
Number of Surgeries
150
100
50
0
High
Medium
Low
1-Jan
29-Jan
26-Feb
25-Mar
22-Apr
20-May
17-Jun
15-Jul
12-Aug
9-Sep
7-Oct
4-Nov
2-Dec

## Ventricular septal defect closure

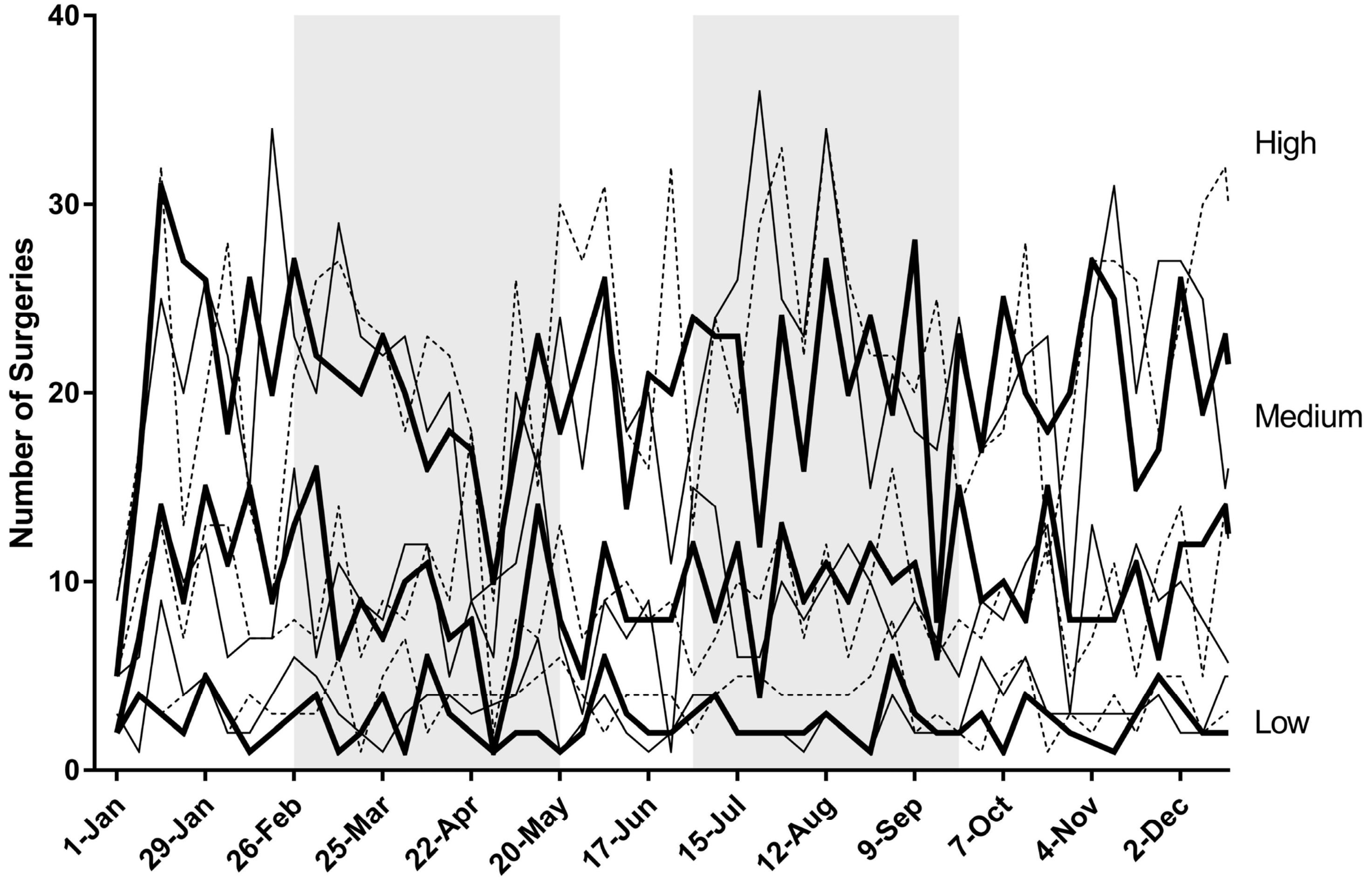


## Resection of mediastinal tumor

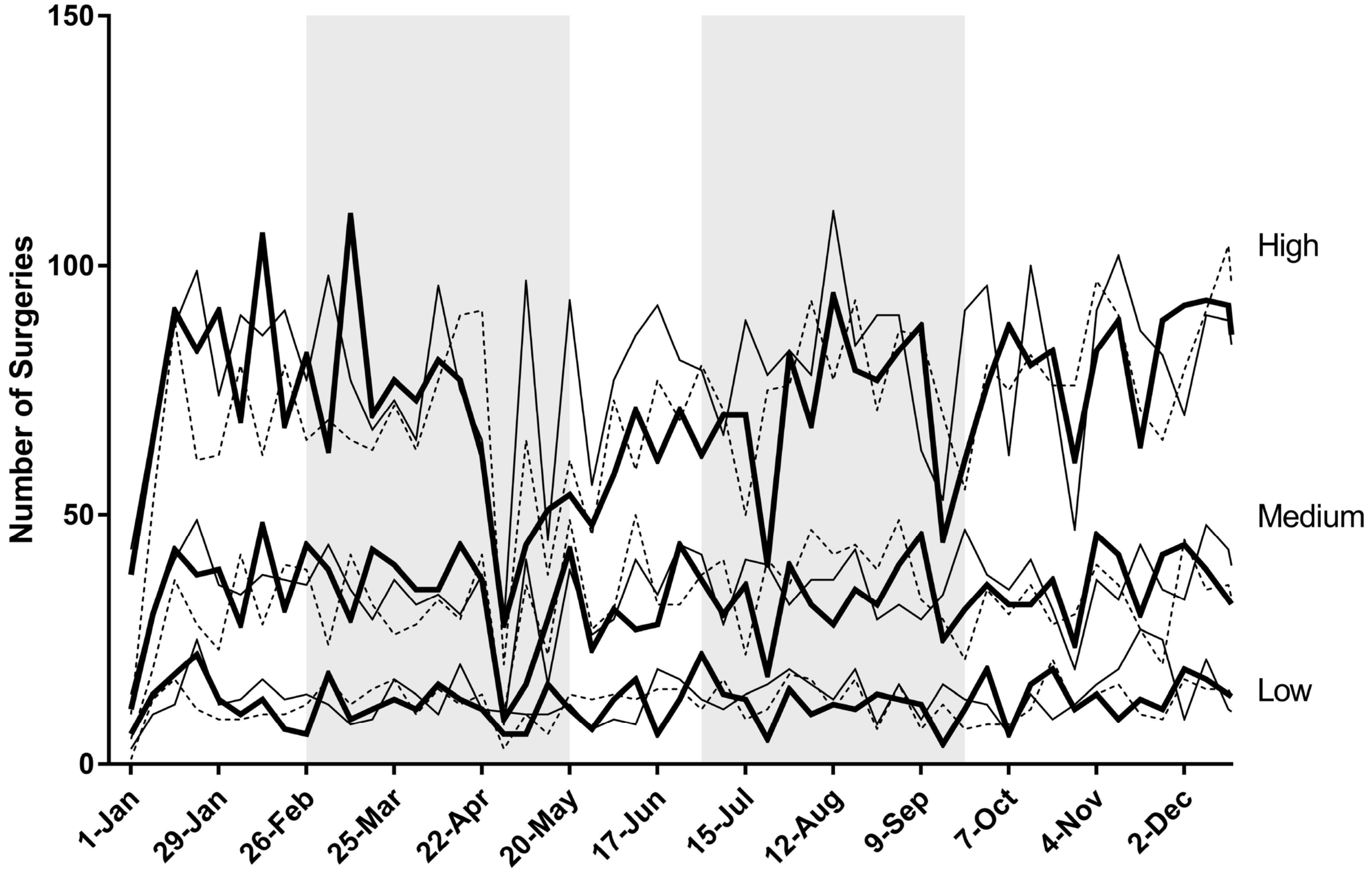

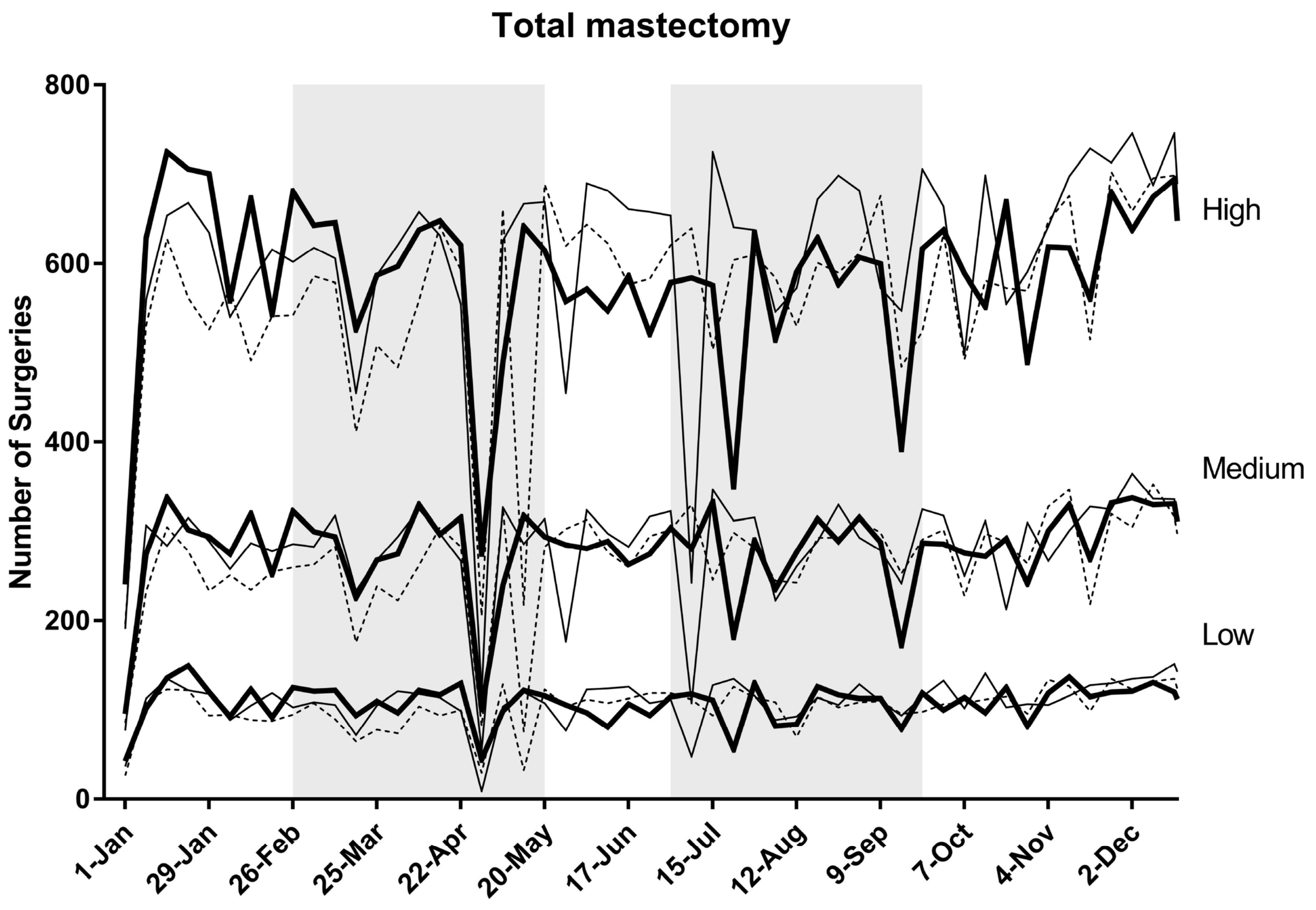

Total mastectomy
800
600
400
200
0
Number of Surgeries
1-Jan
29-Jan
26-Feb
25-Mar
22-Apr
20-May
17-Jun
15-Jul
12-Aug
9-Sep
7-Oct
4-Nov
2-Dec
High
Medium
Low


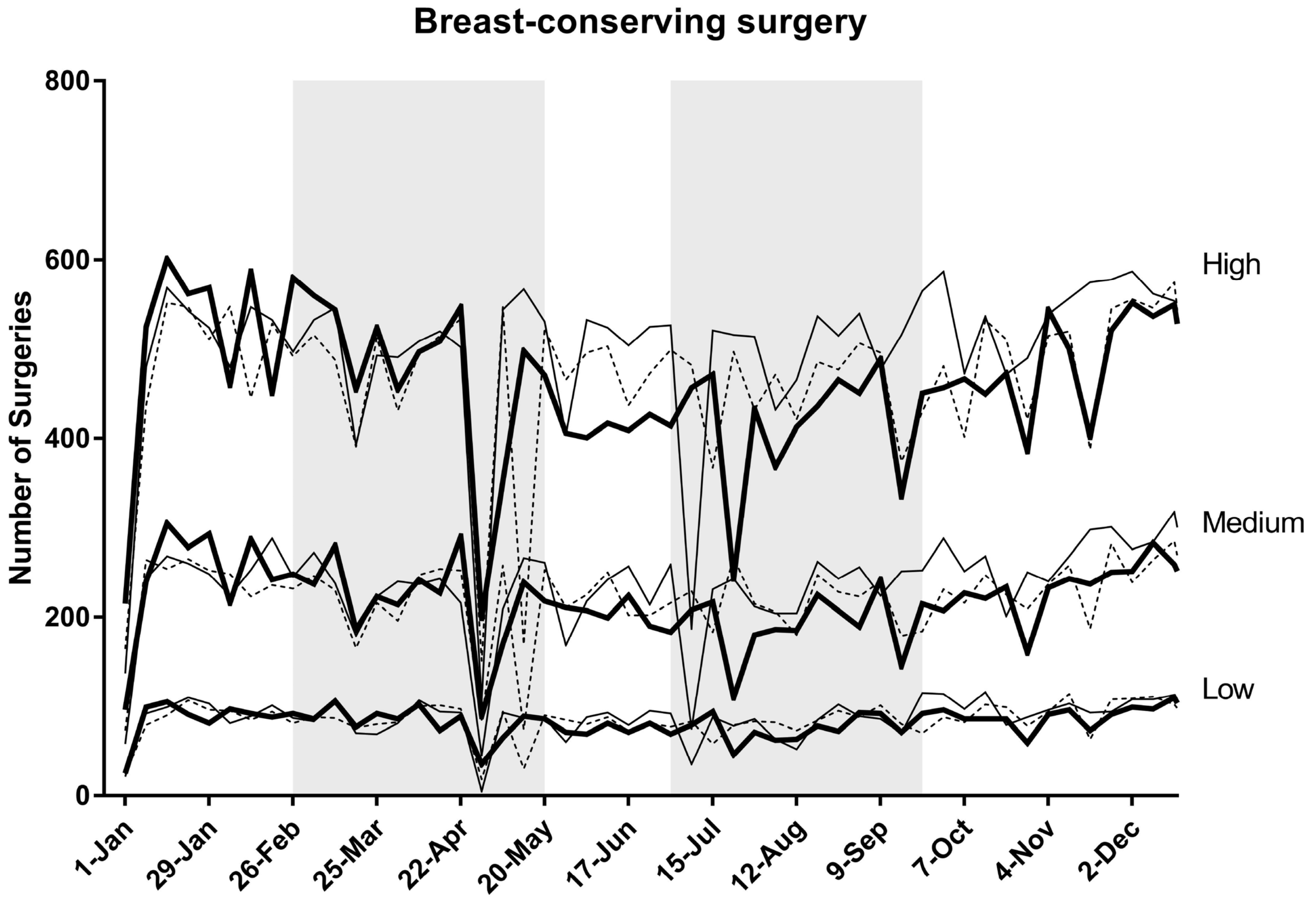

Breast-conserving surgery
800
600
400
200
0
Number of Surgeries
1-Jan
29-Jan
26-Feb
25-Mar
22-Apr
20-May
17-Jun
15-Jul
12-Aug
9-Sep
7-Oct
4-Nov
2-Dec
High
Medium
Low

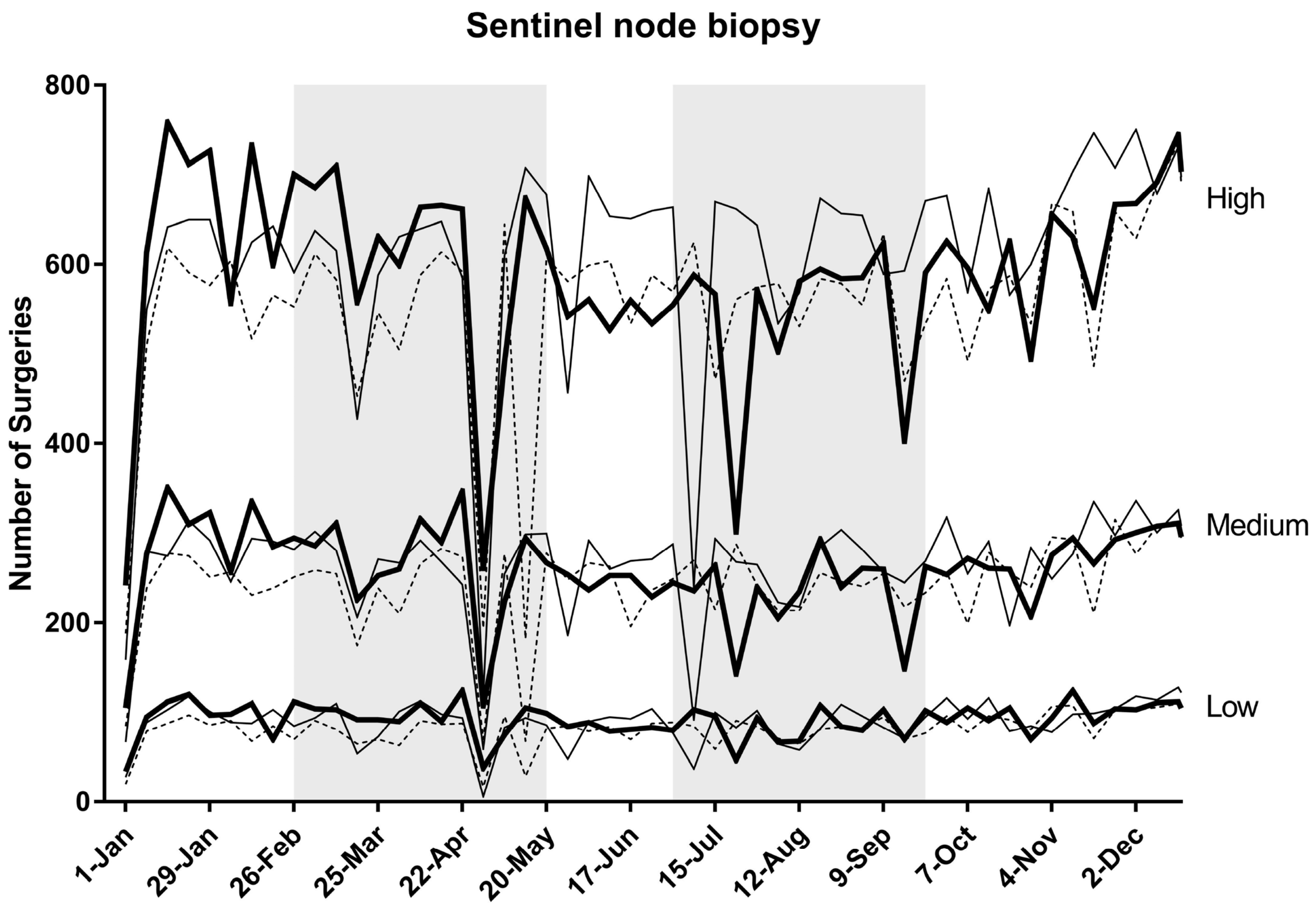

Sentinel node biopsy
Number of Surgeries
800
600
400
200
0
High
Medium
Low
1-Jan
29-Jan
26-Feb
25-Mar
22-Apr
20-May
17-Jun
15-Jul
12-Aug
9-Sep
7-Oct
4-Nov
2-Dec


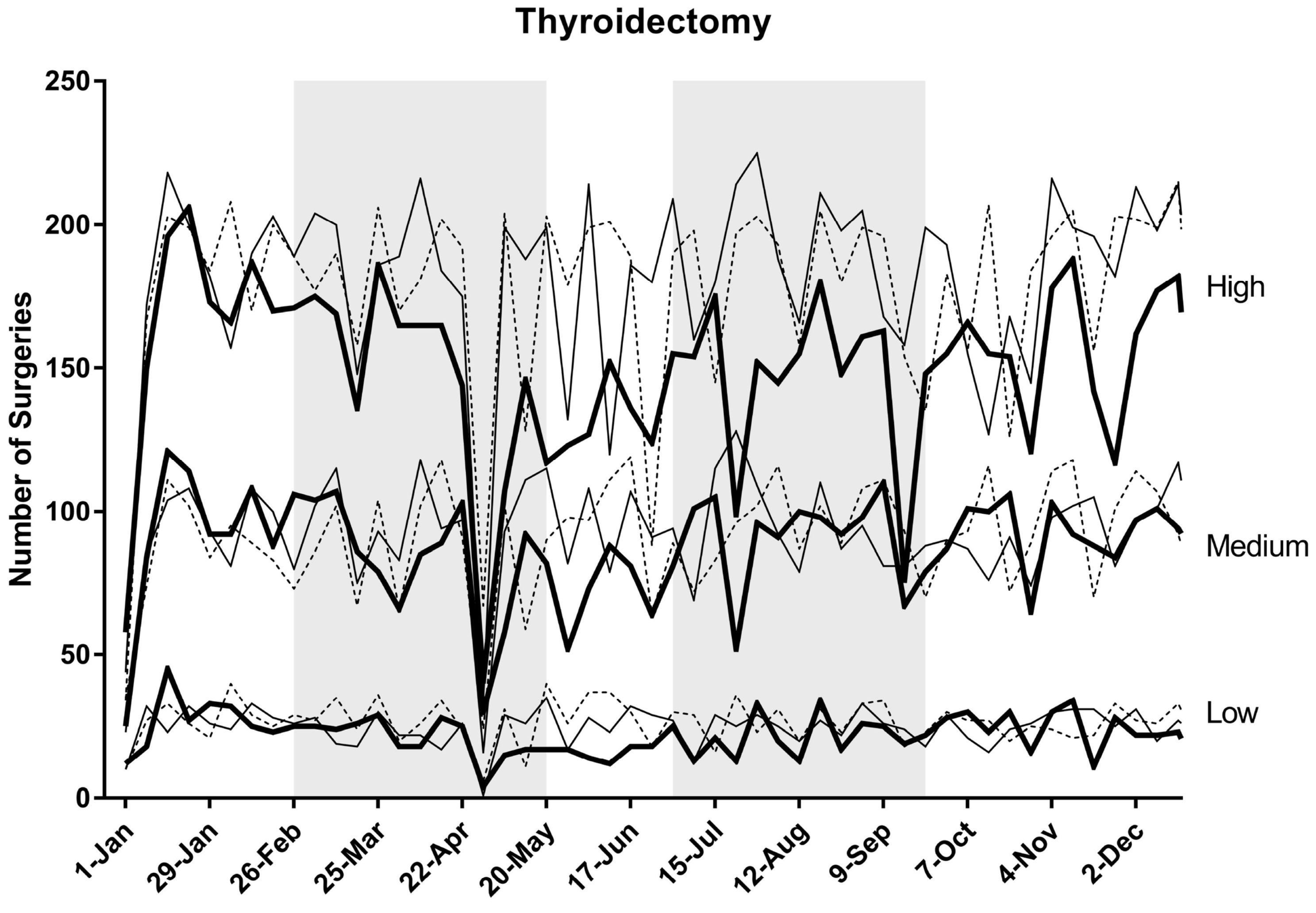

Thyroidectomy
Number of Surgeries
250
200
150
100
50
0
High
Medium
Low
1-Jan
29-Jan
26-Feb
25-Mar
22-Apr
20-May
17-Jun
15-Jul
12-Aug
9-Sep
7-Oct
4-Nov
2-Dec

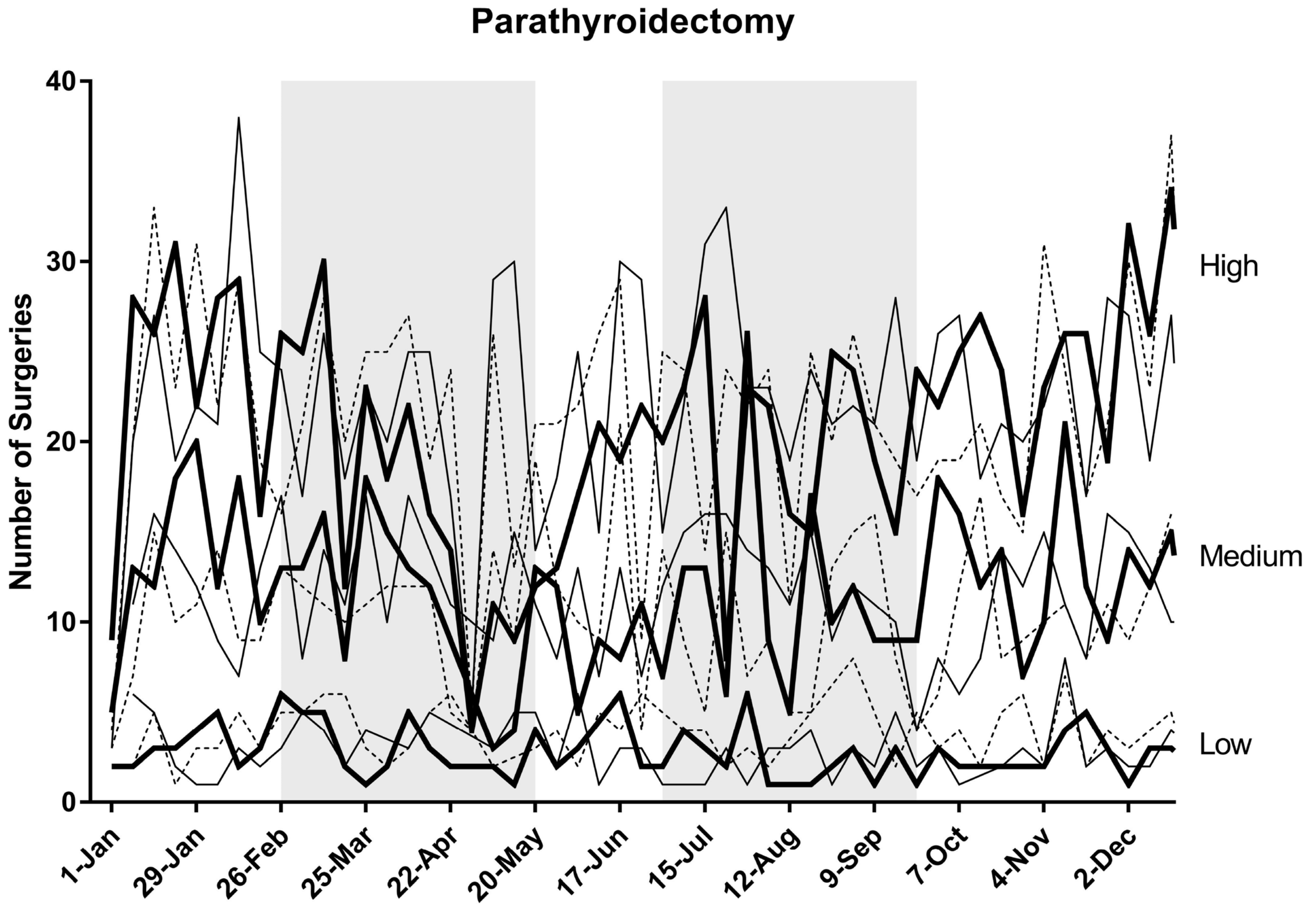
Parathyroidectomy
Number of Surgeries
40
30
20
10
0
High
Medium
Low
1-Jan
29-Jan
26-Feb
25-Mar
22-Apr
20-May
17-Jun
15-Jul
12-Aug
9-Sep
7-Oct
4-Nov
2-Dec

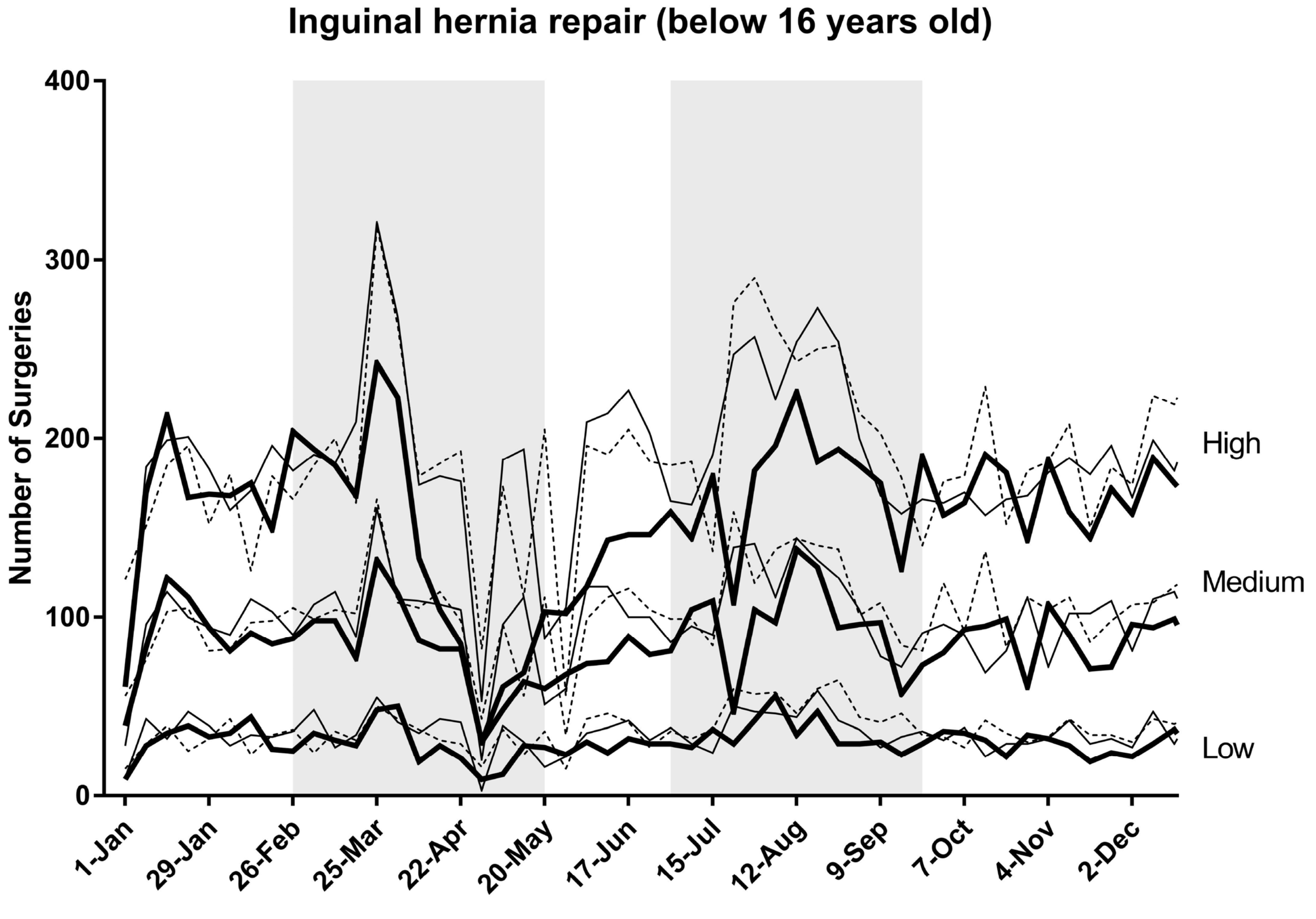
Inguinal hernia repair (below 16 years old)
Number of Surgeries
400
300
200
100
0
High
Medium
Low
1-Jan
29-Jan
26-Feb
25-Mar
22-Apr
20-May
17-Jun
15-Jul
12-Aug
9-Sep
7-Oct
4-Nov
2-Dec

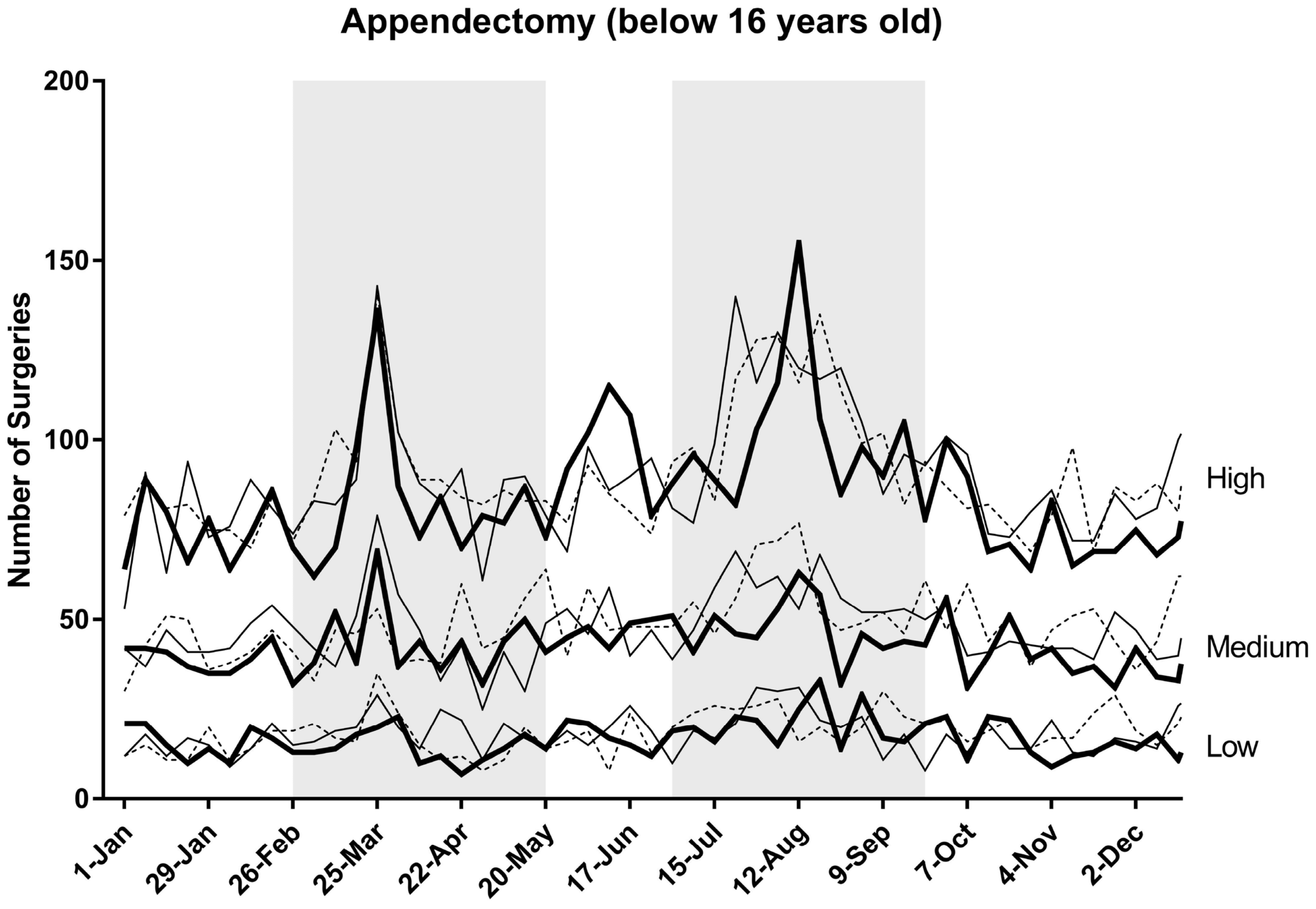

Appendectomy (below 16 years old)
Number of Surgeries
200
150
100
50
0
High
Medium
Low
1-Jan
29-Jan
26-Feb
25-Mar
22-Apr
20-May
17-Jun
15-Jul
12-Aug
9-Sep
7-Oct
4-Nov
2-Dec
